# PRRSV NSP5 orchestrates dual immune disruption by targeting NLRP3 and STING

**DOI:** 10.1186/s13567-025-01636-3

**Published:** 2025-10-16

**Authors:** Xiangyu Huang, Xuyan Xiang, Xiaohan Jiang, Weiyu Qu, Yufei Zhang, Zhenchao Zhao, Minjie Li, Haiwei Wang, Xin Li

**Affiliations:** 1 State Key Laboratory of Veterinary Public Health and Safety, Key Laboratory of Animal Epidemiology of the Ministry of Agriculture and Rural Affairs, College of Veterinary Medicine, China Agricultural University, Beijing, 100193 China; 2 State Key Laboratory of Animal Disease Control, Harbin Veterinary Research Institute, Chinese Academy of Agricultural Sciences, Harbin, 150069 China

**Keywords:** PRRSV, NSP5, NLRP3 inflammasome, STING, IFN-I

## Abstract

Inflammasomes and interferons are two critical defense mechanisms of innate immunity, and their imbalance is a key strategy employed by viruses to evade host immune surveillance. During porcine reproductive and respiratory syndrome virus (PRRSV) infection, an arteritis virus, a characteristic “high inflammation, low interferon” immune imbalance is observed. This study identifies PRRSV non-structural protein NSP5 as a central mediator of this immune imbalance. We demonstrate that NSP5 recruits NLRP3 to the endoplasmic reticulum (ER)-mitochondrial interface, triggering ER stress and Ca^2+^ leakage, which subsequently activates the NLR family pyrin domain containing 3 (NLRP3) inflammasome. Notably, a specific NSP5 mutation (G30A) abolishes its ability to activate NLRP3. PRRSV carrying this mutation exhibits suppressed NLRP3 activation and IL-1β release. Concurrently, NSP5 retains STING in the ER, preventing its trafficking and inhibiting type I interferon induction. This dual mechanism ultimately drives the high inflammation and low interferon phenotype observed during PRRSV infection. Importantly, our findings reveal that a single viral protein can orchestrate immune imbalance, suggesting a potentially widespread strategy for viral evasion of host immune surveillance.

**Supplementary Information:**

The online version contains supplementary material available at 10.1186/s13567-025-01636-3.

## Introduction

The inflammasome and type I interferon (IFN-I) pathways are two fundamental innate immune mechanisms that protect the host against a broad range of microbial pathogens. Inflammasomes are multiprotein complexes that sense intracellular danger signals, typically mediated by effector molecules like NLRP3 [1]. These complexes recognize pathogen-associated molecular patterns (PAMPs) or damage-associated molecular patterns (DAMPs) [2]. The activation of the NLRP3 inflammasome occurs in two stages [1, 3]. The priming stage involves activators such as TLRs that induce NF-κB activation, upregulating the transcription and translation of inflammasomes-related genes (e.g., NLRP3, Casp1, and Pro-IL-1β) [4]. In the activation stage, NLRP3 detects a stimulus and nucleates an inflammasome complex with apoptosis-associated speck-like protein containing a caspase recruitment domain (ASC) and Pro-caspase-1, leading to caspase-1 activation, which drives the secretion of IL-1β and IL-18 and triggers pyroptosis, a form of programmed inflammatory cell death [5–7]. NLRP3 is uniquely activated by diverse and seemingly unrelated stimuli, including ionic imbalances (e.g., K^+^ or Ca^2+^), lysosomal rupture, mitochondrial dysfunction, and Golgi fragmentation [8–11].

Conversely, the IFN-I signaling pathway represents another essential arm of innate immunity that protects against viral infections. The stimulator of interferon genes (STING) serves as a key regulator of DNA-induced IFN-I production [12–14]. STING is activated by 2′ 3′-cGAMP, a second messenger synthesized by the cytosolic DNA sensor cyclic GMP-AMP synthase (cGAS) [15, 16]. Once activated, STING translocates from the endoplasmic reticulum (ER) to the perinuclear region, including the Golgi apparatus, where it phosphorylates the TANK-binding kinase 1 (TBK1), which then phosphorylates the interferon regulatory factor 3 (IRF3) [17–19]. Phosphorylated IRF3 undergoes dimerization, nuclear translocation, and binding to the IFN-I promoter, initiating IFN-I transcription [20]. Secreted IFN-I binds to its receptor IFNAR, triggering the expression of numerous interferon-stimulated genes (ISGs) that interfere with various stages of viral replication [21], ultimately establishing a robust antiviral state within cells. Emerging evidence indicates that the cGAS-STING pathway plays a pivotal role in immune defense against RNA viruses [22–25].

Although inflammasome and IFN-I pathways have traditionally been considered independent, growing evidence reveals significant crosstalk between them [26–30]. IFN-I suppresses NLRP3 inflammasome activation via signal transducer and activator of transcription 1 (STAT1)-dependent mechanisms or through ISGs such as cholesterol 25-hydroxylase (ch25h), which produces 25-hydroxycholesterol (25-HC) to inhibit sterol regulatory element binding protein (SREBP) processing, thereby reducing IL-1β transcription and inflammasome activation [31]. Conversely, inflammasomes also regulate IFN-I responses. Inflammasome activation triggers caspase-1-mediated degradation of cGAS, thereby suppressing IFN-I production [29]. Additionally, IL-1β released by inflammasomes upregulates suppressor of cytokine-signaling-1 (SOCS1), which inhibits MyD88-IRF7 signaling, further dampening IFN-I expression [30].

Immune imbalance is a hallmark of viral infections, enabling viruses to evade immune surveillance [32, 33]. In SARS-CoV-2 infection, patients exhibit delayed and diminished IFN-I responses alongside excessive pro-inflammatory cytokine release [34–36]. A similar phenomenon of “excessive inflammation and aberrant IFN-I responses” has been observed during porcine reproductive and respiratory syndrome virus (PRRSV), an arteritis virus, infection [37–39], yet the precise molecular mechanisms remain unclear. Since its emergence in the late 1980s, PRRSV has posed a major threat to global pig production [40, 41]. This positive-sense RNA virus actively suppresses host immunity, dampens cytotoxic T cell responses, and evades sterilizing immunity induced by current vaccines [42, 43]. These immune evasion strategies allow PRRSV to persist in infected animals, facilitating the emergence of immune-escape mutants and causing substantial economic losses in the global swine industry [44–46].

Here, we report that the non-structural protein NSP5 of PRRSV plays a dual role in activating inflammasomes and suppressing IFN-I signaling. Mechanistically, NSP5 interacts with the nucleotide-binding and oligomerization (NACHT) domain of NLRP3, recruiting it to the endoplasmic reticulum (ER)-mitochondria, where it induces ER stress and calcium leakage, leading to the NLRP3 inflammasome activation. Notably, a specific mutation in NSP5 (G30A) completely abolishes its ability to activate NLRP3. PRRSV carrying this mutation exhibits significantly reduced NLRP3 activation and IL-1β release. Simultaneously, NSP5 interacts with STING in the ER, preventing its translocation to the Golgi apparatus, thereby suppressing IFN-I production. These findings shed light on PRRSV immune evasion strategies, demonstrating how a single viral protein orchestrates immune imbalance by activating inflammasomes while inhibiting antiviral interferon responses.

## Materials and methods

### Ethics statement

Porcine Alveolar Macrophages (PAMs) were isolated following approved protocols, and animal challenge experiments were approved by the Committee on the Ethics of Animal Experiments of the Harbin Veterinary Research Institute (HVRI) of the Chinese Academy of Agricultural Sciences (CAAS) and the Animal Ethics Committee of Heilongjiang Province, China. The license numbers associated with this research protocol were 231,017–01-GR and 241,022–04-GR.

### Cell lines and virus

HEK-293T cells, iPAMs were cultured in Dulbecco’s Modified Eagle Medium (DMEM) (MACGENE, #CM10013). Marc-145 cells were cultured in RPMI 1640 medium. Porcine Alveolar Macrophages (PAMs) were isolated from 4-week-old specific pathogen-free (SPF) piglets and cultured in RPMI 1640 medium. All cells were supplemented with 10% fetal bovine serum (FBS) (PlantChemMed, #PC-00001), 100 U/mL penicillin, and 100 μg/mL streptomycin (Solarbio, #P1400), and maintained at 37 °C in a 5% CO_2_ incubator. iPAMs were a gift from Professor Yandong Tang at the Harbin Veterinary Research Institute (China).

Highly pathogenic porcine reproductive and respiratory syndrome virus (HP-PRRSV, GenBank accession no: PV067901) was isolated and preserved in the Laboratory of Harbin Veterinary Research Institute.

### Generation and characterization of PRRSV mutants

The HP-PRRSV infectious clone was double digested with XhoI and NdeI. Site-directed mutations in the PRRSV NSP5 gene, specifically G30A, N35X (X represents the 20 essential AA.), and I33V, were introduced into the digested infectious clone. These mutants were confirmed by sequencing. After 3–5 days post-infection, the supernatants were collected and seeded into a 96-well plate. The supernatants were then collected and saved after a series of passages. The cells were washed three times with PBS, fixed with 4% paraformaldehyde for 30 min at room temperature, and permeabilized with 0.1% Triton X-100 for 15 min at room temperature. Each step was followed by three washes with PBS. The cells were incubated with primary antibodies at 37 °C for 1 h and rinsed three times. Alexa Fluor 488 Goat anti-mouse IgG (H + L) secondary antibodies were used for 1 h at room temperature, and then the cells were observed under a digital inverted microscope.

### Plasmids and transfection

DNAs encoding swine NLRP3, swine Caspase-1, swine IL-1β, swine IRF3 and swine NEK7 were cloned into the pCAGGS or pcDNA3.1( +) vectors. DNAs encoding swine ASC and STING were cloned into pEGFP-C1 vectors. Plasmids encoding Flag-tagged HP-PRRSV structural and non-structural proteins were constructed by our laboratory. For the truncated forms of NLRP3, the PCR products were inserted into pcDNA3.1( +) with an HA tag. To screen for key sites in NSP5 that activate the NLRP3 inflammasome, DNA encoding PRRSV NSP5 wild-type (WT), 1-58aa, 86-170aa, 59-170aa, 1-122aa, 1-58aa, 59-122aa, 13-170aa, 1-143aa, FLL15AAA, WRM18AAA, MGH21AAA, WTP25AAA, LVAV29AAAA, GFF32AAA, ILN35AAA, EIL38AAA, PAVL42AAAA, VRS45AAA, VFS48AAA, FGM51AAA, FVL54AAA, SWLT58AAAA, I33A, I33V, G30S, G30A, and N35A were cloned into the pcDNA3.1-mCherry vector. A plasmid encoding RR-mNeonGreen was a gift from Dr. Shengda Xie (Nanjing Agricultural University). All the plasmids were confirmed by DNA Sanger sequencing. According to the manufacturer’s protocol, transient transfection of the above plasmids was performed using Lipofectamine 2000 (Thermo Fisher Scientific, #11668500) or Lipofectamine 3000 (Thermo Fisher Scientific, #L3000015).

### Reagents and antibodies

LPS (HY-D1056), Nigericin (HY-127019), Pan-Caspase inhibitor Z-VAD (HY-16658) and MCC950 (HY-12815) were obtained from MedChemExpress. EGTA (S4598), 2-Aminoethyl diphenylborinate (2-APB) (S6657), BAPTA-AM (S7534), Dantrolene sodium (S5478) and diABZI (S8796) were obtained from Selleck. Swine IL-1β ELISA KIT (SEKP-0001) was purchased from Solarbio.

The following antibodies were used in this study: PRRSV Nucleocapsid antibody (GTX129270) from GeneTex; PRRSV M (3F7) mAb in our laboratory; HA tag (51,064–2-AP) and Flag tag (20,543–1-AP) antibodies from Proteintech; CoraLite488- and CoraLite594-conjugated Goat Anti-Rabbit IgG (SA00013-2, SA00013-4) and CoraLite488- and CoraLite594-conjugated Goat Anti-Mouse IgG (SA00013-1, SA00013-3) from Proteintech; Goat anti-Mouse IgG (H + L) Cross-Adsorbed Secondary Antibody, Alexa Fluor™ 633 (A-21050) from Thermo Fisher; β-actin (sc-47778) and ASC (sc-514414) antibodies from SantaCruz; NLRP3 (WL02635) and Caspase-1 (WL02996a) antibodies from WanleiBio; Flag tag (F1804) antibody from Sigma; HA tag (3724S), STING (13,647), TBK1(3504), p-TBK1(5483), IRF3 (4302) antibodies from Cell Signaling Technology; GSDMD (ab155233) and p-IRF3 (ab182859) antibodies from Abcam.

### Hematoxylin and eosin staining and immunohistochemistry

Piglet lung tissues were isolated and fixed in 10% formalin at room temperature for 24 h. After fixation, pathological sectioning was performed by Servicebio.

### Co-immunoprecipitation and western blotting

After washing the cells with 1 × PBS, the cells were lysed in NP-40 buffer on ice for 30 min. The supernatant was collected after centrifugation, with one-fifth reserved as input. The remaining sample was incubated with Protein A/G and IgG antibodies at 4 °C for 4–6 h, followed by overnight incubation with Flag antibody and Protein A/G at 4 °C. The samples were washed, and 80 μL of NP-40 buffer with 5 × SDS loading buffer was added for western blot analysis.

For western blot analysis, protein samples were transferred to PVDF membranes (0000223060, Immobilon). After blocking with 5% skim milk in PBST (0.01 M phosphate-buffered saline, pH 7.2, 0.05% Tween 20) at room temperature for 2 h, the membranes were incubated overnight with a monoclonal antibody at 4 °C. After washing, the membranes were incubated with anti-mouse/rabbit IgG HRP-Linked antibodies (7076/7074, Cell Signaling Technology) at 37 °C for 1 h. Chemiluminescent detection of the target proteins on the membranes was performed using a chemiluminescence detection kit (170–5061, BIO-RAD).

### Ca^2+^ measurements

The Fluo-8 Calcium Flux Analysis Kit (Abcam, ab112129) was used to measure cytoplasmic Ca^2+^ levels. Briefly, at the specified time points, 100 μL of Fluo-8 dye-loading solution was added to each well of a 96-well plate containing 1 × 10^5^ cells per well. The plate was incubated for 30 min at 37 °C, followed by another 30 min at room temperature. Calcium flux was assessed by measuring fluorescence with excitation/emission wavelengths of 490/525 nm.

### RNA isolation and quantitative real-time PCR (RT-qPCR)

RNA was extracted from whole-cell lysates using the RNA Simple Total RNA Kit (TIANGEN, #DP419) and then reverse transcribed to cDNA using the HiScript II Q RT SuperMix for qPCR (+ gDNA wiper) Kit (Vazyme, #R223-01). Quantitative PCR (qPCR) was performed with the ChamQ SYBR qPCR Master Mix (Vazyme, #Q712-02). The threshold cycle values were normalized to GAPDH, using triplicate samples amplified with specific primers for GAPDH.

### Cytotoxicity and IL-1β detection

Cell death was assessed using the CytoTox 96 Non-Radioactive Cytotoxicity Assay (Promega, #G1780). In a 96-well plate, 50 μL of cell culture supernatant was combined with 50 μL of LDH assay buffer and incubated at 37 °C for 30 min. The reaction was terminated by adding the stop solution, and absorbance was determined using a microplate reader (Bio-Rad).

For measuring IL-1β release, cell culture supernatants were collected and analyzed using the Porcine IL-1β ELISA Kit (Solarbio, #SEKP-0001).

### ASC-speck-based system

HEK-293 T cells were seeded into a 24-well plate (5 × 10^5^ cells per well) and incubated overnight. pcDNA3.1-NLRP3-HA (50 ng), pEGFP-C1-ASC (5 ng), pCAGGS-Caspase-1 (3 ng), pcNDA3.1-Pro-IL-1β (200 ng) were co-transfected with 50 ng PRRSV structural or non-structural proteins. For positive control, HEK-293T cells were transfected for 12 h, exposed to LPS (100 ng/mL) for 8 h, and then treated with nigericin for 4 h. Cells were observed using fluorescence microscopy (Evos FL Auto2 fluorescence microscope).

### Confocal microscopy

After infection or transfection, the cells were fixed with 4% paraformaldehyde at room temperature for 15 min. Following fixation, the cells were permeabilized with 0.1% Triton X-100. To assess the co-localization, the cells were stained with specified antibodies. Images were captured and analyzed using a Nikon A1 confocal microscope (Japan).

### Statistical analysis

The duplicate experimental samples were subjected to t-test analysis to determine the means ± standard deviation (SD) using GraphPad Prism 9.5 software. Significance levels were denoted as follows: * for *P* < 0.05, ** for *P* < 0.01, and *** for *P* < 0.001. ns for no significance.

## Results

### PRRSV infection induces pyroptosis and IL-1β release

Highly pathogenic PRRSV (HP-PRRSV) infection in pigs is frequently associated with a significant release of inflammatory cytokines, particularly IL-1β, while antiviral IFN-I levels remain abnormally low [37–39]. This immune imbalance, characterized by “excessive inflammation but insufficient antiviral response” suggests that PRRSV actively manipulates host immune responses through complex mechanisms, leading to immune dysfunction.

**Figure 1:**
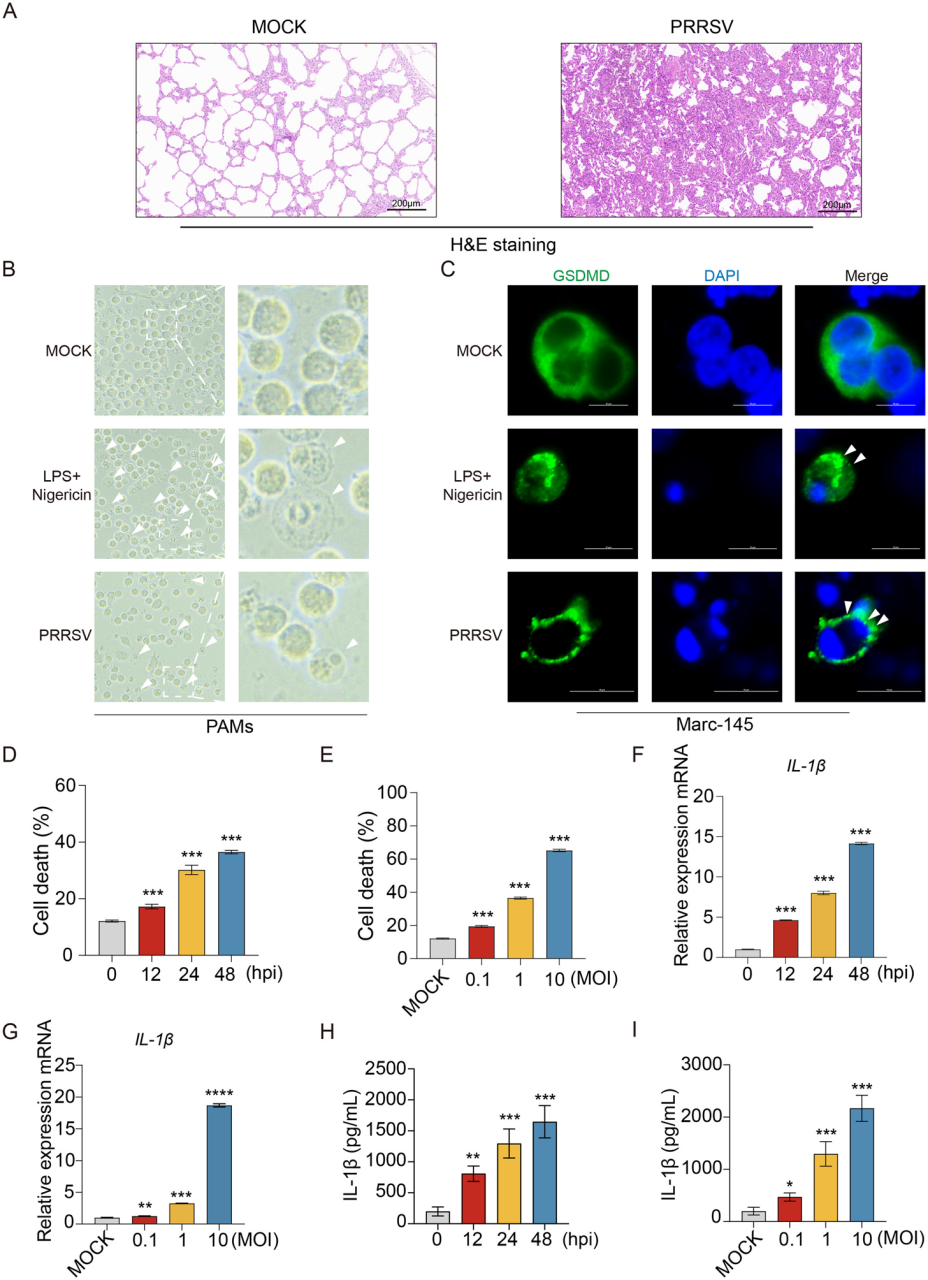
**PRRSV infection induces pyroptosis and IL-1β release.** **A** Representative images of lungs from mock-infected control piglets and piglets infected with porcine reproductive and respiratory syndrome virus (PRRSV). Lung sections were stained with hematoxylin and eosin (H&E). Scale bars = 200 μm. **B** Porcine alveolar macrophages (PAMs) were infected with PRRSV at an multiplicity of infection (MOI) of 1 for 12 h. PAMs were treated with LPS for 8 h followed by nigericin for 4 h as positive controls. The cell morphology of PAMs was observed using an Evos FL Auto2 fluorescence microscope (white arrows, pyroptotic cells). Scale bars = 10 μm. **C** Confocal microscopy analysis of GSDMD distribution in Marc-145 cells following PRRSV infection or LPS and Nigericin treatment. GSDMD was labeled with an anti-GSDMD antibody (green), and nuclei were stained with DAPI (blue). Arrows indicate GSDMD dots. Scale bars = 10 μm. **D** to **I** PAMs were infected with PRRSV at different times or at various MOIs. LDH-release-based cell death measurement in the cell culture supernatants was detected by the LDH assay kit (D and E). Swine *IL-1β* mRNA levels (related to swine GAPDH) were analyzed by quantitative RT-PCR (qPCR) (F and G). Swine IL-1β levels in the cell culture supernatants were detected by ELISA (H and I). The *p* value of less than 0.05 was considered statistically significant. * for *p* < 0.05, ** for *p* < 0.01, *** for *p* < 0.001, ns for not significant.

Pyroptosis is an inflammatory and lytic form of programmed cell death, triggered by inflammasome assembly, caspase-1 activation, and GSDMD cleavage, ultimately causing plasma membrane rupture and the release of inflammatory cytokines such as IL-1β and IL-18 [3, 47]. To investigate whether PRRSV infection induces pyroptosis in vivo and in vitro, we examined HP-PRRSV-infected lung tissues from infected pigs. Histopathological analysis revealed significant microscopic lesions, including marked alveolar septal thickening and lymphocyte infiltration, compared to the mock-infected control group (Figure 1A).

To further validate these findings, we isolated primary porcine alveolar macrophages (PAMs) from Specific Pathogen Free (SPF) pigs and challenged them with PRRSV, alongside Marc-145 cells, a well-established PRRSV-permissive cell line. PRRSV-infected PAMs exhibited distinct pyroptotic morphological changes, including cell rounding, swelling, and the characteristic “fried egg” appearance due to nuclear protrusion, similar to the effects observed in cells treated with LPS and nigericin (Figure 1B). In Marc-145 cells, GSDMD localization was diffusely distributed in uninfected cells, whereas PRRSV infection or nigericin treatment induced punctate GSDMD aggregates near the plasma membrane, further confirming pyroptosis activation (Figure 1C). To assess PRRSV-induced pyroptosis quantitatively, we infected PAMs with varying MOIs or at different time points (Additional files 1A and B) and measured lactate dehydrogenase (LDH) release, a marker of cell death. Consistent with the morphological observations, LDH release was significantly elevated in PRRSV-infected PAMs in a time- and dose-dependent manner (Figures 1D and E). Concurrently, the *IL-1β* mRNA transcription and IL-1β protein secretion were markedly increased in a time- and dose-dependent manner (Figures 1F–I), along with other inflammatory cytokines, IL-6 and TNF-α (Additional files 1C-1F). Collectively, these results demonstrate that PRRSV infection induces pyroptosis and drives excessive inflammation in vitro and in vivo.

### PRRSV infection activates NLRP3 inflammasome

**Figure 2:**
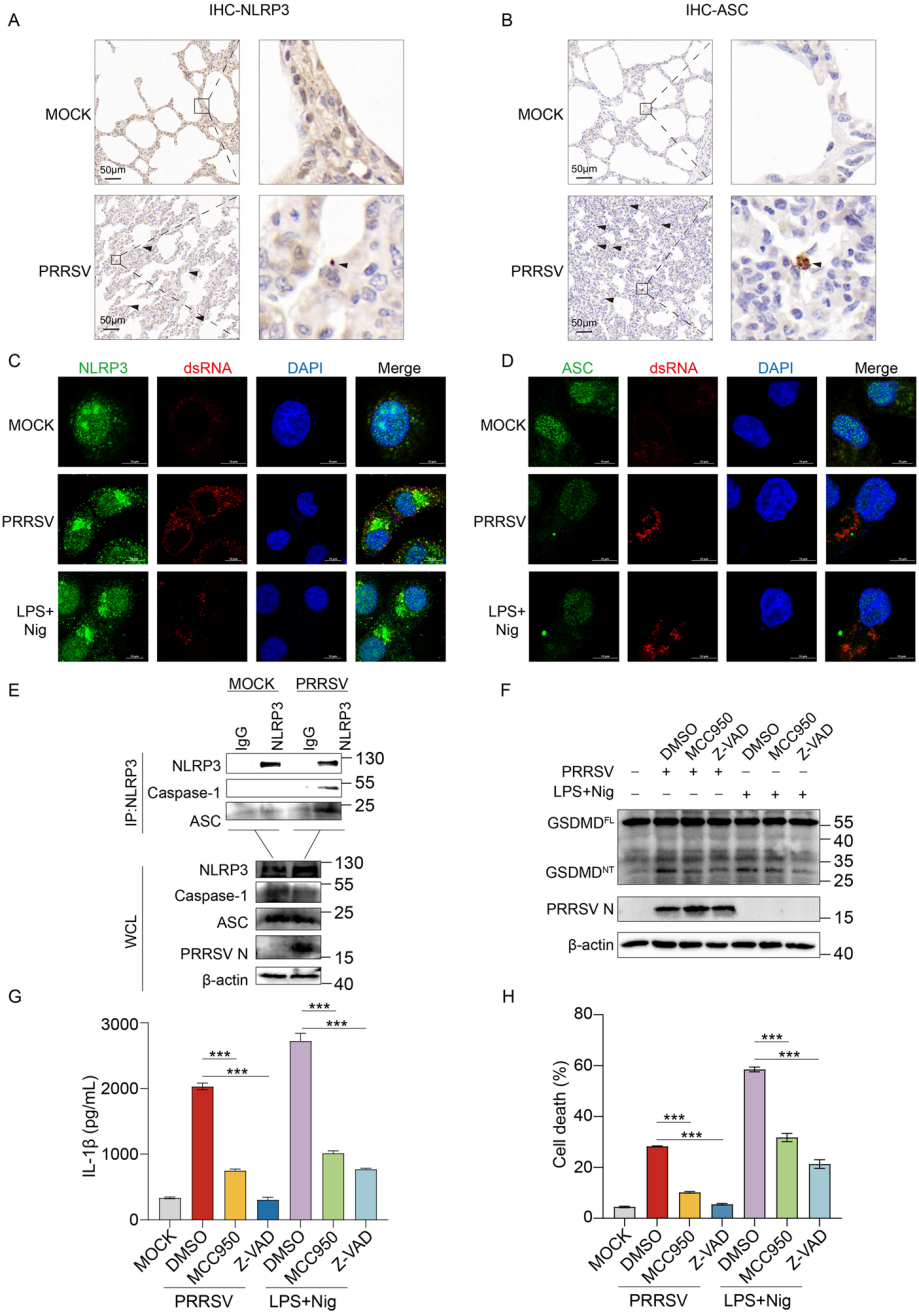
**PRRSV infection activates NLRP3 inflammasome.** **A** Representative images of ASC immunohistochemical (IHC) staining in lung sections from mock-infected and PRRSV-infected piglets. Arrows indicate NLRP3 aggregates. **B** Representative images of ASC IHC staining in lung sections from mock-infected and PRRSV-infected piglets. Arrows indicate ASC Specks. Scale bars = 50 μm. **C** and **D** Marc-145 cells were infected with PRRSV at an MOI of 1 for 36 h. Marc-145 cells were treated with LPS for 8 h followed by nigericin for 4 h as positive controls. Confocal microscopy analysis of NLRP3 aggregates in Marc-145 cells following PRRSV infection or LPS/Nigericin treatment. NLRP3 was labeled with an anti-NLRP3 antibody (green), ASC was labeled with an anti-ASC antibody (green), PRRSV infection was labeled with anti-dsRNA (Red) and nuclei were stained with DAPI (blue). Scale bars = 10 μm. **E** Marc-145 cells were infected with PRRSV (MOI = 1) for 36 h. Anti-NLRP3 antibody or isotype control IgG was used for immunoprecipitation, followed by immunoblotting for NLRP3, Caspase-1 and ASC. **F** to **H** PAMs were pretreated with MCC950 or Z-VAD for 2 h before being infected with PRRSV (MOI = 1) for 24 h or stimulated with LPS and Nigericin. Cell lysates were analyzed by western blotting for GSDMD, PRRSV-N, and β-actin (**F**). Swine IL-1β levels in the cell culture supernatants were detected by ELISA (**G**). LDH-release-based cell death measurement in the cell culture supernatants was detected by LDH assay kit (**H**). The *p* value of less than 0.05 was considered statistically significant. *** for *p* < 0.001.

Previous studies have shown that PRRSV-induced pyroptosis and inflammation are mediated by the NLRP3 inflammasome [48]. To further investigate whether HP-PRRSV activates pyroptosis and inflammation via the NLRP3 inflammasome, we examined its activation in vitro and in vivo. To assess NLRP3 inflammasome activation in vivo, we performed immunohistochemical analysis on lung tissue sections from PRRSV-infected and uninfected piglets. Compared to the uninfected control group, PRRSV-infected lung tissue exhibited increased aggregation of NLRP3 and the formation of ASC specks, indicating inflammasome activation (Figures 2A and B).

In uninfected cells, NLRP3 exhibited a diffuse distribution. Upon stimulation with LPS and nigericin, NLRP3 aggregated in the cytoplasm, which was also observed following PRRSV infection (Figure 2C). Similarly, ASC, an inflammasome adaptor protein, displayed a diffuse distribution in resting cells but formed characteristic "ASC specks" following LPS and nigericin stimulation (Figure 2D). Notably, PRRSV infection also induced ASC speck formation, further confirming inflammasome activation (Figure 2D). ASC oligomerization, a direct indicator of inflammasome activation, was detected 24 to 48 h post-PRRSV infection (Additional file 2A). Upon activation, NLRP3 recruits ASC, which subsequently recruits Caspase-1 to assemble the inflammasome complex. To verify this process, we performed co-immunoprecipitation using NLRP3 antibody or isotype control IgG to detect endogenous NLRP3-ASC-Caspase-1 complex. Upon PRRSV infection, the NLRP3-ASC-Caspase-1 complex was clearly detected by the immunoprecipitation of NLRP3 antibody, whereas no such complex was observed with the isotype control IgG or uninfected cells (Figure 2E).

To further confirm the critical role of the NLRP3 inflammasome in PRRSV-induced pyroptosis and inflammation, PAMs were pretreated with the NLRP3 inhibitor MCC950 and the pan-caspase inhibitor Z-VAD before PRRSV infection or LPS and nigericin stimulation. Both MCC950 and Z-VAD treatments significantly suppressed PRRSV-induced GSDMD cleavage, IL-1β secretion, and LDH release, consistent with findings in the positive control group treated with LPS and nigericin (Figures 2F–H). Taken together, these findings show that PRRSV infection activates the NLRP3 inflammasome, leading to pyroptosis and inflammatory responses.

### PRRSV NSP5 is sufficient to trigger the NLRP3 inflammasome

**Figure 3:**
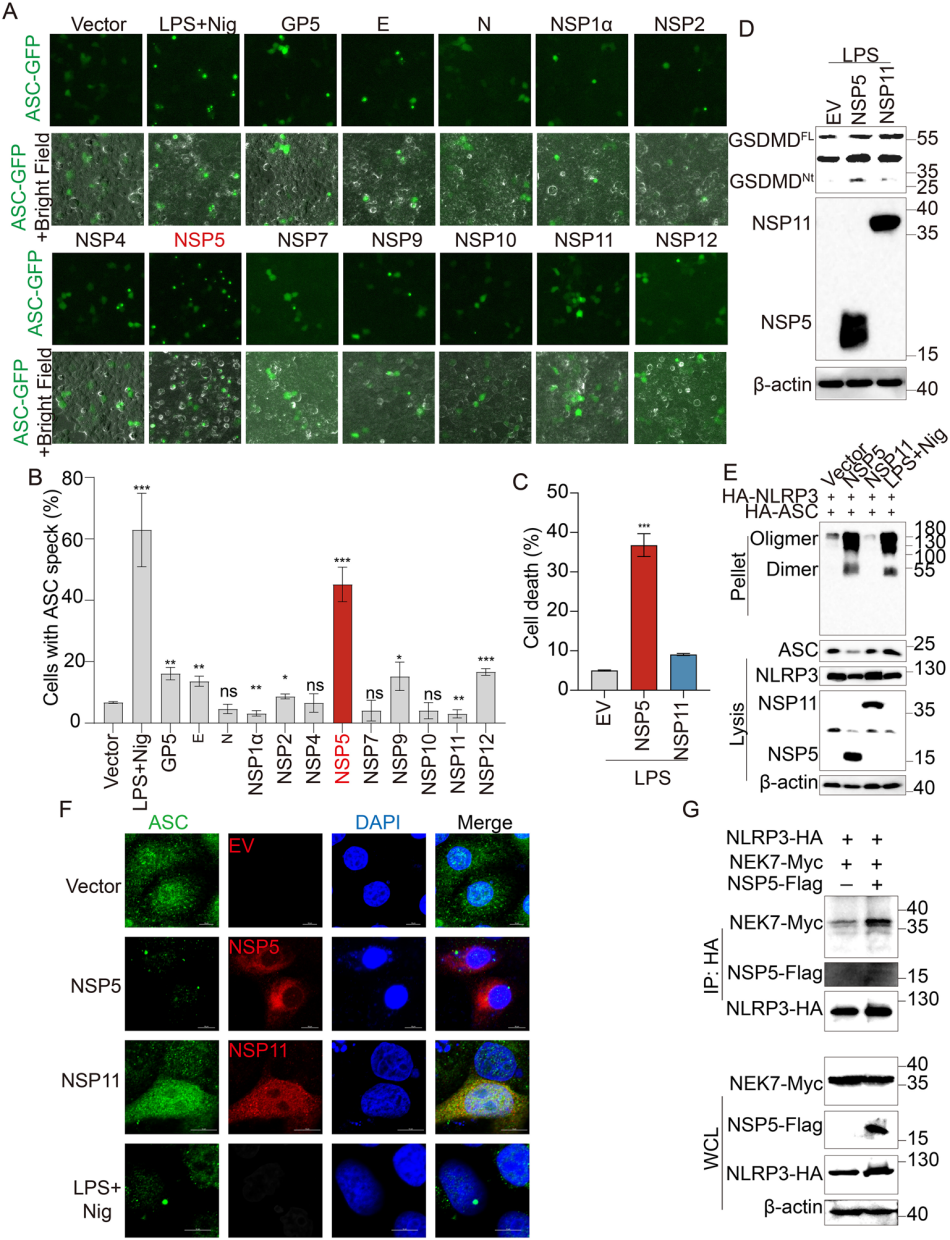
**PRRSV NSP5 is sufficient to trigger NLRP3 inflammasome.** **A** and **B** HEK-293T cells were transfected with a plasmid encoding one of the PRRSV structural or no structural proteins in the presence of sNLRP3 inflammasome system (50 ng pCAGGS-sNLRP3-HA, 5 ng pEGFP-C1-ASC, 3 ng pCAGGS-Caspase-1-HA, 200 ng pcDNA3.1-Pro-IL-1β-HA), ASC specks were observed using fluorescence microscopy. ASC specks were visualized using fluorescence microscopy (**A**) and quantified (**B**). **C** and **D** LPS-primed Marc-145 cells were transfected with a plasmid encoding NSP5 or NSP11. Supernatants and cell lysates were collected 36 h post-transfection (hpt) for LDH assay (**C**) and western blotting using the indicated antibodies (**D**). **E** and **F** Marc-145 cells were transfected with plasmids encoding sNLRP3-HA, sASC-HA and NSP5-Flag or NSP11-Flag, followed by LPS treatment. Cells treated with LPS for 8 h followed by nigericin for 4 h as positive controls. **E** 36 hpt, the cell lysates were prepared, and the pellets were washed with PBS for three times and cross-linked using DSS for western blotting. **F** 36 hpt, the cells were then fixed and probed with anti-ASC (green) and anti-Flag (red) antibodies, and nucleus marker DAPI (blue), and then observed by confocal microscopy. Scale bars = 10 μm. **G** HEK-293 T cells were co-transfected with plasmids encoding sNLRP3-HA and Myc-sNEK7 along with Flag-NSP5 or empty vector. Anti-HA antibody was used for immunoprecipitation, followed by immunoblotting. The *p* value of less than 0.05 was considered statistically significant. * for *p* < 0.05, ** for *p* < 0.01, *** for *p* < 0.001, ns for not significant. The experimental data are representative of results from three independent experiments.

To determine whether PRRSV-encoded proteins activate the NLRP3 inflammasome, we screened 12 PRRSV-encoded proteins via ASC-speck-based system, because ASC-speck formation is a hallmark of inflammasome assembly [49, 50]. The screening results revealed that GP5, E, NSP2, NSP5 and NSP12 induced ASC-speck formation, with NSP5 exhibiting the strongest activation effect (Figures 3A and B). Notably, GP5 has been previously reported to activate NLRP3 [51], validating the reliability of our screening system (Figures 3A and B).

Due to the low transfection efficiency in PAMs, we overexpressed NSP5 in Marc-145 cells and measured LDH release and GSDMD cleavage, using NSP11 as a negative control. The results showed that NSP5 expression significantly increased LDH release and GSDMD cleavage, whereas NSP11 and the empty vector group showed no significant effect (Figures 3C and D). In the ASC oligomerization assay, NSP5 strongly induced ASC oligomerization, whereas NSP11 and the empty vector group showed minimal oligomerization (Figure 3E). Similarly, endogenous ASC-Speck formation was markedly increased in NSP5-expressing cells, compared to the positive group with LPS and nigericin stimulation, whereas NSP11 and the empty vector group exhibited no ASC-speck formation (Figure 3F and Additional file 2B). Moreover, a mitosis-related serine/threonine kinase, NEK7 (NIMA-related kinase), has been reported to interact with NLRP3, playing a crucial role in NLRP3 activation [52, 53]. To investigate whether NSP5 enhances the NLRP3-NEK7 interaction, we co-transfected HEK-293 T cells with NLRP3 and NEK7, along with either NSP5 or an empty vector as a negative control, and performed co-immunoprecipitation assays. The results showed that the interaction between NLRP3 and NEK7 was significantly enhanced in the presence of NSP5 (Figure 3G). Collectively, these findings demonstrate that PRRSV NSP5 is sufficient to activate the NLRP3 inflammasome by inducing GSDMD cleavage, ASC oligomerization, ASC-speck formation, and enhancing the NLRP3-NEK7 interaction.

### PRRSV NSP5 interacts with the NACHT domain of NLRP3

**Figure 4:**
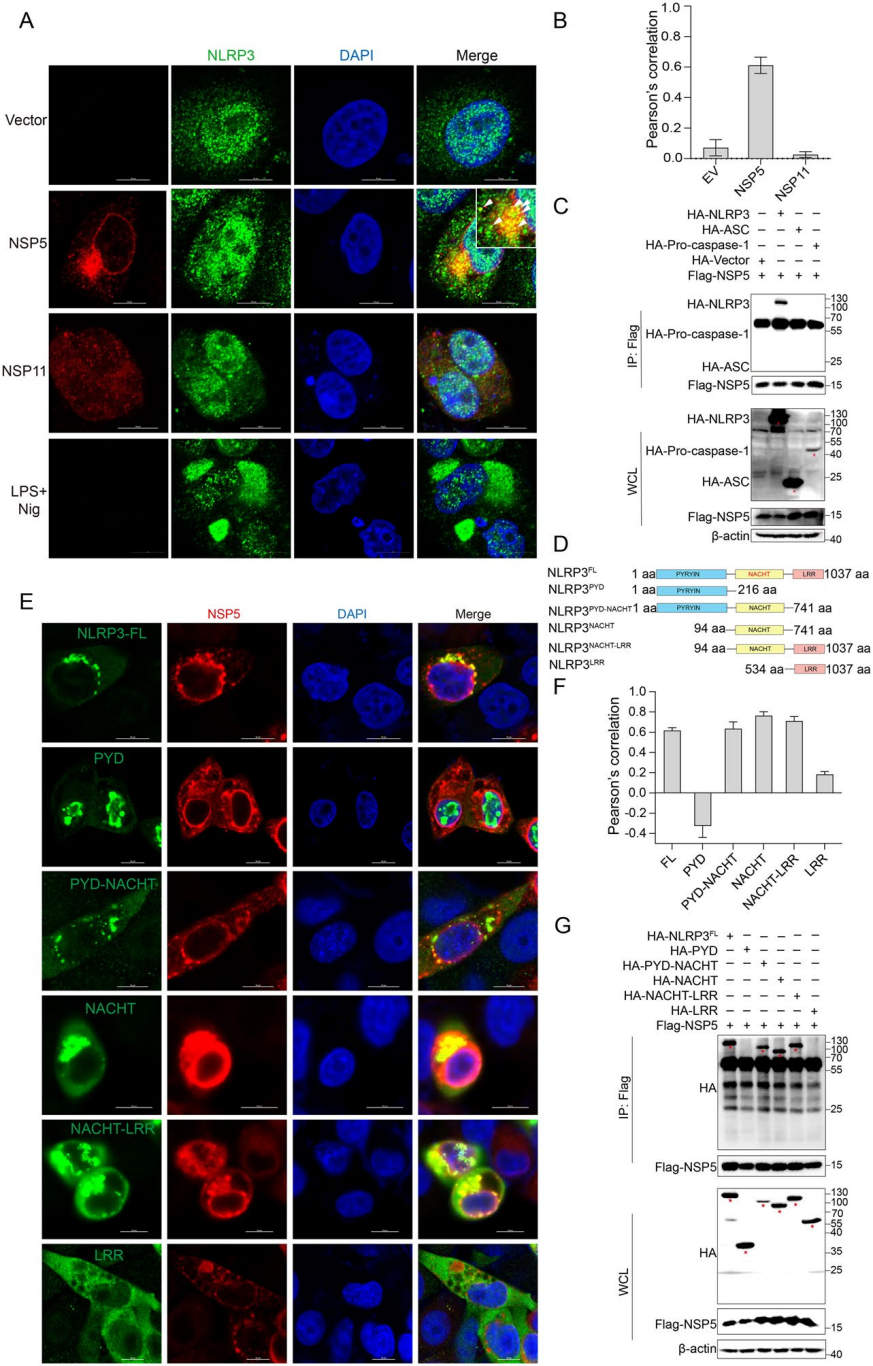
**PRRSV NSP5 interacts with the NACHT domain of NLRP3.** **A** and **B** Marc-145 cells were transfected with a plasmid encoding NSP5-Flag or NSP11-Flag. Cells treated with LPS for 8 h followed nigericin for 4 h as positive controls. At 36 hpt, the cells were then fixed and probed with anti-NLRP3 (green) and anti-Flag (red) antibodies, and nucleus marker DAPI (blue), and observed by confocal microscopy (b). Scale bars = 10 μm. The colocalization analysis was expressed as Pearson’s correlation coefficient. Results are represented as the mean ± SD of data from three independent experiments (**B**). **C** HEK-293T cells were transfected with a plasmid encoding NSP5-Flag along with a plasmid encoding NLRP3-HA, Caspase-1-HA or ASC-HA. At 36 hpt, the cells were lysed and whole cell lysates (WCL) were immunoprecipitated with anti-Flag mAb. The immunoprecipitants were detected by immunoblotting with the antibodies indicated. **D** Schematic representation of the full-length NLRP3 and its truncated mutants. **E** and **F** Marc-145 cells were transfected with a plasmid encoding NSP5-Flag along with HA-NLRP3 or its deletion mutants. At 36 hpt, the cells were then fixed and probed with anti-HA (green) and anti-Flag (red) antibodies, and nucleus marker DAPI (blue), and then observed by confocal microscopy (**E**). Scale bars = 10 μm. The colocalization analysis was expressed as Pearson’s correlation coefficient. Results are represented as the mean ± SD of data from three independent experiments (**F**). **G** HEK-293T cells were transfected with a plasmid encoding NSP5-Flag along with a plasmid encoding NLRP3-HA or its deletion mutants. At 36 hpt, the cells were lysed and whole cell lysates (WCL) were immunoprecipitated with anti-Flag mAb. The immunoprecipitants were detected by immunoblotting with the antibodies indicated.

The activation of the NLRP3 inflammasome typically involves two key stages: the priming stage and the activation stage [5, 6]. To determine whether NSP5 plays a role in the priming stage, we performed qPCR analysis to measure *nlrp3* mRNA transcription levels. The results showed that NSP5 overexpression did not alter the mRNA levels of *nlrp3* (Additional files 3A and B), suggesting that NSP5 likely functions during the activation stage. To further investigate the role of NSP5 in inflammasome activation, we examined its subcellular localization using confocal microscopy. The results revealed that NSP5 co-localized with endogenous NLRP3, whereas NSP11 did not (Figures 4A and B). Next, we sought to determine whether NSP5 interacts with other components of the NLRP3 inflammasome complex, including ASC and Caspase-1. Co-immunoprecipitation (Co-IP) assays demonstrated that NSP5 specifically interacted with NLRP3 but not with ASC or Caspase-1 (Figure 4C). To further map the specific domain of NLRP3 required for NSP5 interaction, we constructed five truncated NLRP3 mutants (Figure 4D) and analyzed them using confocal microscopy and Co-IP assays. Confocal microscopy results showed that NSP5 co-localized with the PYD-NACHT, NACHT, and NACHT-LRR domains but not with the PYD or LRR domains (Figures 4E and F). Similarly, Co-IP results confirmed that NSP5 specifically co-precipitated with the PYD-NACHT, NACHT, and NACHT-LRR domains but not with the PYD or LRR domains (Figure 4G). Taken together, these results indicate that the NACHT domain of NLRP3 is the critical region mediating its interaction with NSP5.

### PRRSV infection induces NLRP3 translocation to the ER and mitochondria

**Figure 5:**
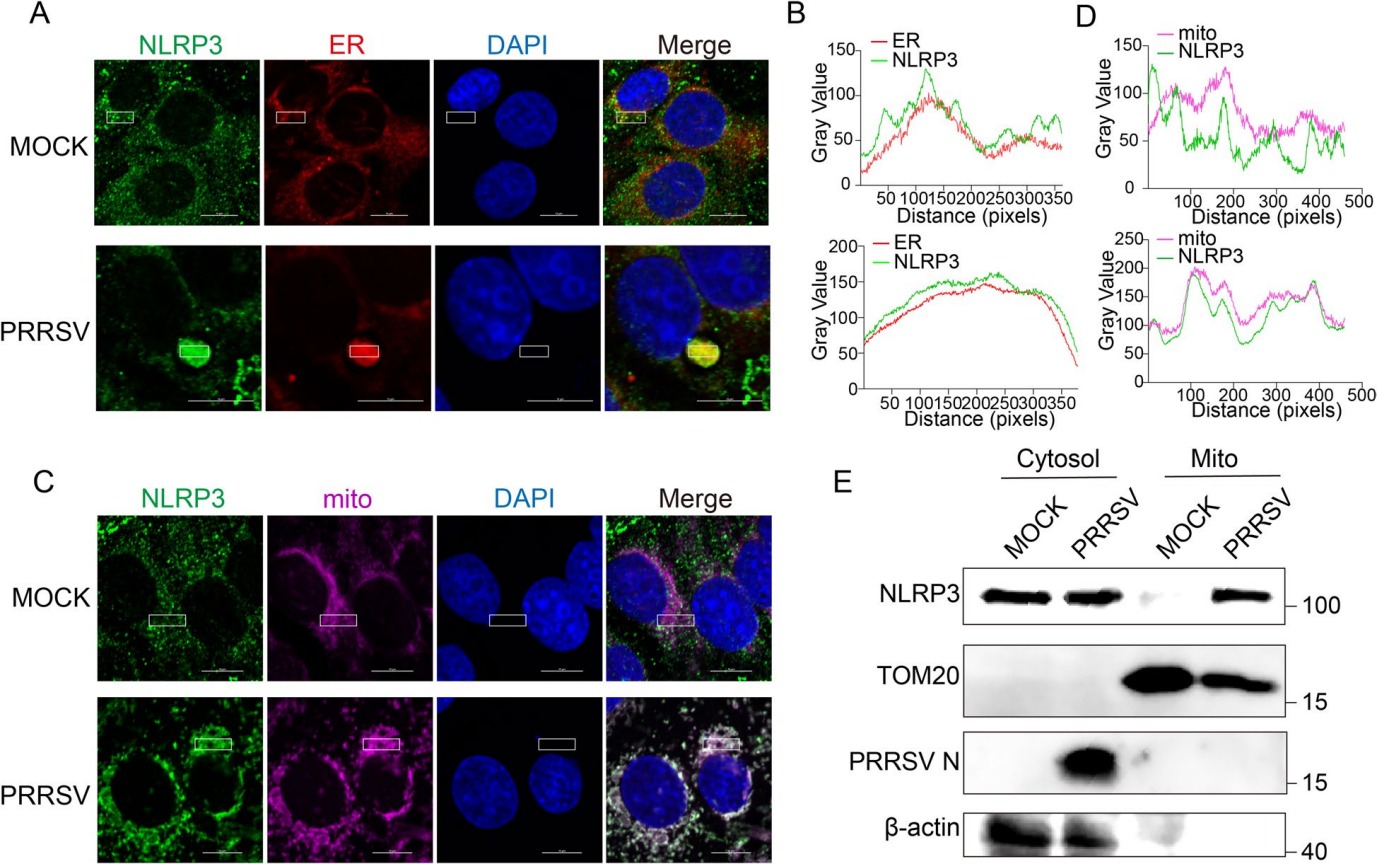
**PRRSV infection induces the translocation of NLRP3 to the ER and mitochondria.** Marc-145 cells were infected with PRRSV at an MOI of 1 for 36 h. The Cells were incubated with an anti-NLRP3 antibody (green) and stained with DAPI and ER-specific fluorescent dye (red) or mitochondria-specific fluorescent dye (pink). **A** and **C** Co-localization of NLRP3 with the ER or mitochondria was analyzed using confocal microscopy following PRRSV infection. Scale bars = 10 μm. **B** and **D** The colocalization of NLRP3 and the ER or mitochondria was analyzed by ImageJ software. Images are representative of 3 biological replicates. **E** The distribution of NLRP3 in the cytosol and mitochondria after PRRSV infection was analyzed. Cytosolic and mitochondrial fractions were separated using a mitochondrial isolation kit, followed by western blotting with antibodies against NLRP3, PRRSV N, β-actin, and TOM20. The experimental data are representative of results from three independent experiments.

Under steady-state condition, NLRP3 is diffusely distributed in the cytoplasm, partially localization to the ER but not to mitochondria or the Golgi apparatus. Upon stimulation, NLRP3 clusters in the ER and mitochondria [54], facilitating NLRP3 inflammasome assembly. To investigate whether PRRSV infection promotes NLRP3 localization to the ER and mitochondria, we infected Marc-145 cells with PRRSV and stained them with ER-specific dyes, mitochondrial dyes, and NLRP3 antibodies. Confocal microscopy analysis revealed that in uninfected cells, NLRP3 was sporadically co-localized with the ER and completely absent from the mitochondria (Figures 5A–D). However, in PRRSV-infected cells, NLRP3 prominently co-localized with both the ER and mitochondria (Figures 5A–D). To further validate the confocal microscopy observations, we performed mitochondrial fractionation experiments to assess NLRP3 localization biochemically. Consistent with our microscopy results, NLRP3 was clearly detectable in mitochondrial fractions upon PRRSV infection, whereas it was nearly absent in uninfected cells (Figure 5E). These findings indicate that PRRSV infection induces the translocation of NLRP3 to the ER and mitochondria.

### PRRSV NSP5 recruits NLRP3 to the ER-Mitochondrial interface to facilitate inflammasome assembly

**Figure 6:**
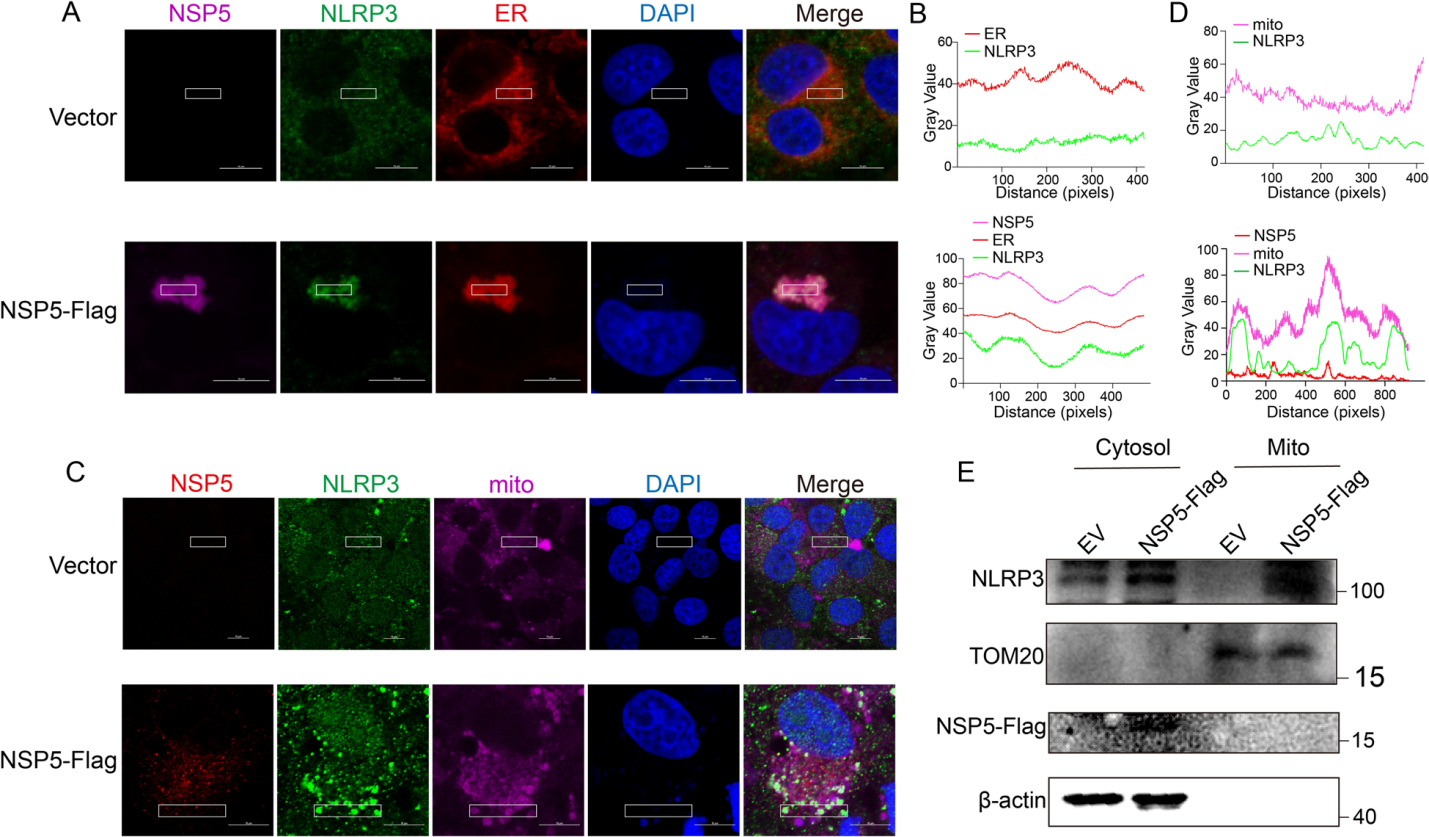
**PRRSV NSP5 recruits NLRP3 to the ER-Mitochondrial interface to facilitate inflammasome assembly.** Marc-145 cells were transfected with a plasmid encoding NSP5-Flag for 36 h. **A** and **C** Co-localization of NLRP3 with the ER or mitochondria was analyzed using confocal microscopy following NSP5 overexpression. Scale bars = 10 μm. **B** and **D** The colocalization was analyzed by ImageJ software. Images are representative of 3 biological replicates. **E** The distribution of NLRP3 in the cytosol and mitochondria following NSP5 overexpression was analyzed. Cytosolic and mitochondrial fractions were separated using a mitochondrial isolation kit, followed by western blotting with antibodies against NLRP3, Flag, β-actin, and TOM20. The experimental data are representative of results from three independent experiments.

NSP5 primarily localizes in the ER, which serves as a key component of the replication and transcription complex (RTC) in PRRSV [55]. The activation and assembly of the NLRP3 inflammasome are closely associated with subcellular organelles, including the ER, mitochondria, and Golgi apparatus. To determine whether NSP5 specifically localizes to the ER upon NLRP3 activation, we overexpressed NSP5 in Marc-145 cells, as suitable endogenous NSP5 antibodies were unavailable. Confocal microscopy revealed that NSP5 predominantly localized to the ER but was absent from mitochondria and Golgi apparatus (Additional files 3C-3H). Our previous results demonstrated that PRRSV infection induces NLRP3 localization to both the ER and mitochondria. Given that NSP5 primarily resides in the ER and interacts with NLRP3, we hypothesized that NSP5 might recruit NLRP3 to the ER. To test this, we overexpressed NSP5 in Marc-145 cells and labeled NLRP3 and ER using NLRP3 antibody and ER-specific fluorescent dye, respectively. In control cells, NLRP3 exhibited a diffuse cytoplasmic distribution with sporadic ER localization, consistent with previous reports [54]. However, in NSP5-expressing cells, NLRP3 aggregated and colocalized with NSP5 and the ER (Figure 6A and B). Since PRRSV infection also promotes NLRP3 translocation to the mitochondria, we further hypothesized that NSP5 expression might facilitate this translocation. In NSP5-overexpressing Marc-145 cells, NLRP3 and mitochondria were labeled with NLRP3 antibody and mitochondrial fluorescent dye, respectively. The results showed that NSP5 expression induced NLRP3 translocation to the mitochondria (Figures 6C and D). To further confirm these findings, mitochondrial fractionation experiments were performed, which revealed that NLRP3 was significantly enriched in mitochondrial fractions following NSP5 overexpression (Figure 6E). These findings demonstrate that during PRRSV infection, NSP5 recruites NLRP3 to both the ER and mitochondria, thereby facilitating the assembly and activation of the NLRP3 inflammasome.

### NSP5 induces ER Ca^2+^ leakage via IP3R

The ER serves as the primary reservoir for intracellular Ca^2+^, and its homeostasis is closely linked to ER stress [56–58]. Depletion of Ca^2+^ within the ER can induce ER stress [59, 60], while excessive cytosolic Ca^2^⁺ flux can directly activate NLRP3 [8, 9] or induce mitochondrial stress by driving excessive Ca^2^⁺ influx into mitochondria [61]. This mitochondrial stress leads to excessive mitochondrial reactive oxygen species (mtROS) production, further contributing to NLRP3 inflammasome activation [62]. These findings collectively suggest that Ca^2+^ leakage serves as a critical signal for NLRP3 inflammasome activation. While NSP5 recruits NLRP3 to the ER-mitochondrial interface and promotes its aggregation, the precise mechanism driving full activation of the NLRP3 inflammasome remains unclear. We hypothesized that NSP5 might trigger Ca^2+^ leakage, thereby activating the NLRP3 inflammasome.

**Figure 7:**
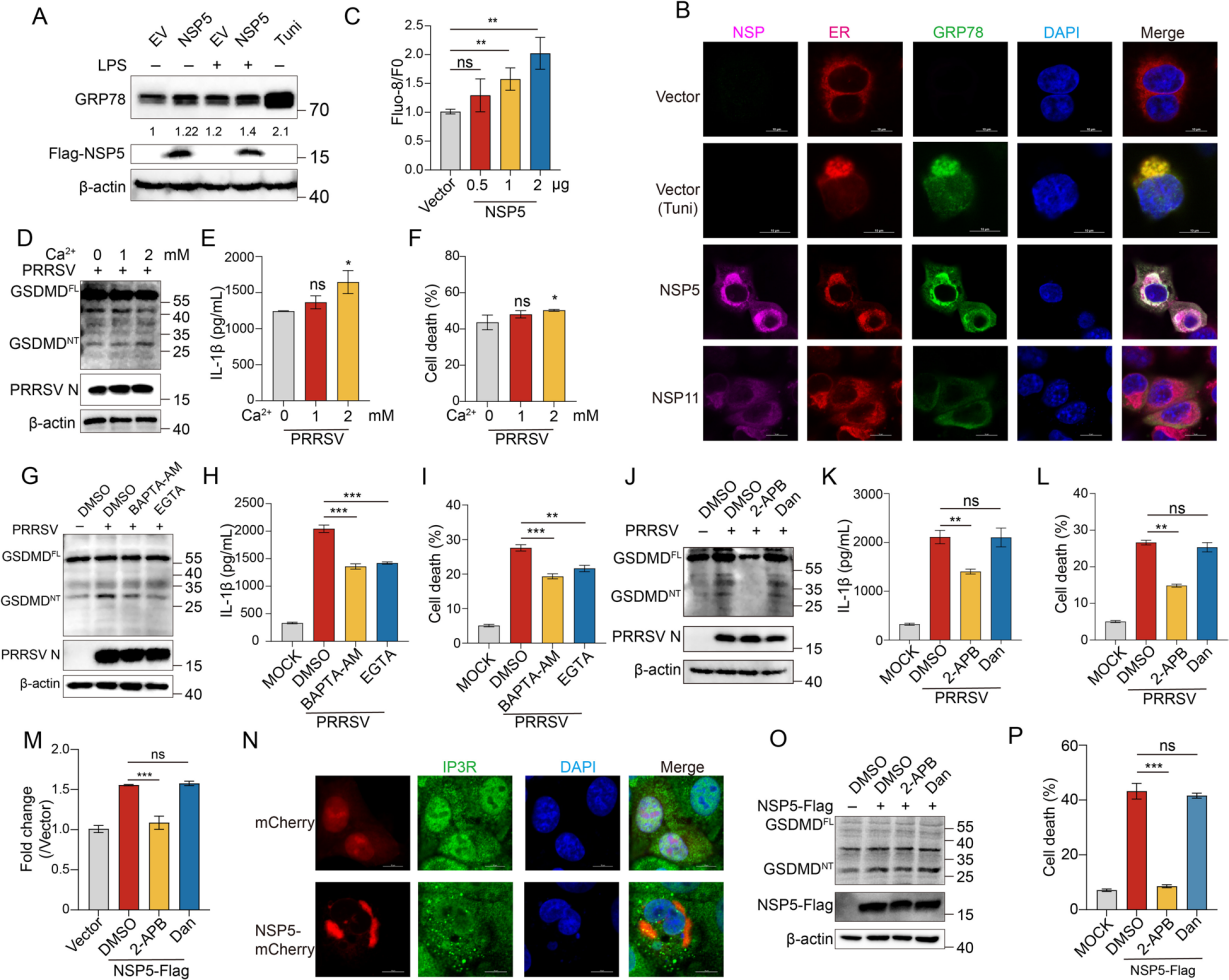
**NSP5 induces ER Ca**^**2+**^** leakage via IP3R.** **A** The effect of NSP5 on ER stress. Marc-145 cells were transfected with a plasmid encoding NSP5-Flag for 36 h under conditions with or without LPS stimulation. **B** Colocalization of NSP5 and GRP78 at ER. Marc-145 cells were transfected with a plasmid encoding NSP5-Flag or NSP11-Flag for 36 h. Marc-145 cells were treated with tunicamycin as control. Scale bars = 10 μm. **C** Marc-145 cells were transfected with pcDNA3.1 or increasing doses of pcDNA-NSP5-Flag for 36 h. Cytoplasmic Ca^2+^ levels were determined by fluorescence of Fluo-8. D to F PAMs were infected with PRRSV (MOI = 1) and cultured in media containing 0, 1, or 2 mM calcium chloride for 24 h. Samples were collected for western blotting (**D**), IL-1β release (**E**), and LDH (**F**) release assays. G to I PAMs were infected with PRRSV (MOI = 1) and treated with DMSO, BAPTA-AM or EGTA for 24 h. Samples were collected for western blotting (**G**), IL-1β release (**H**), and LDH (**I**) release assays. J to L PAMs were infected with PRRSV (MOI = 1) and treated with DMSO, 2-APB or dantrolene sodium for 24 h. Samples were collected for western blotting (**J**), IL-1β release (**K**), and LDH (**L**) release assays. M, O, P Marc-145 cells were transfected with a plasmid encoding NSP5-Flag and treated with DMSO, 2-APB or dantrolene sodium for 36 h. Cytoplasmic Ca^2+^ were determined by fluorescence of Fluo-8 (**M**). Samples were collected for western blotting (**O**) and LDH (**P**) release assays. **N** Co-localization of NSP5 with IP3R was analyzed using confocal microscopy. Scale bars = 10 μm. The *p* value of less than 0.05 was considered statistically significant. * for *p* < 0.05, ** for *p* < 0.01, *** for *p* < 0.001, ns for not significant.

To determine whether NSP5 induces ER stress, we analyzed the expression of glucose-regulated protein 78 (GRP78), a key ER stress marker [63]. Overexpression of NSP5 in Marc-145 cells induced ER stress, as evidenced by increased GRP78 expression, regardless of LPS stimulation (Figure 7A). Tunicamycin-treated cells served as a positive control (Figure 7A). Confocal microscopy showed low GRP78 fluorescence intensity in cells overexpressing an empty vector or NSP11, whereas cells expressing NSP5 exhibited significantly enhanced GRP78 fluorescence, indicating increased ER stress, similar to the effect observed with tunicamycin treatment (Figure 7B). Moreover, NSP5 co-localized with GRP78 in the ER (Figure 7B), further confirming its role in inducing ER stress. Additionally, NSP5 expression was associated with increased cytosolic Ca^2+^ flux (Figure 7C). However, whether PRRSV-induced NLRP3 inflammasome activation is directly dependent on Ca^2+^ signaling remains unclear.

Intracellular Ca^2+^ originates from both extracellular and intracellular sources. We observed that increasing Ca^2+^ flux in calcium-free medium enhanced PRRSV-induced GSDMD cleavage, LDH release, and IL-1β secretion (Figures 7D and F). Conversely, treatment with EGTA, an extracellular Ca^2+^ chelator, significantly suppressed these effects in PRRSV-infected PAMs. Similarly, BAPTA-AM, an intracellular Ca^2+^ chelator, also reduced PRRSV-induced GSDMD cleavage, LDH release, and IL-1β secretion (Figures 7G–I), indicating that both intracellular and extracellular Ca^2+^ are essential for PRRSV-induced NLRP3 inflammasome activation.

Previous studies have shown that PRRSV-induced ER Ca^2+^ leakage is mediated by IP3R channels [51, 64]. To determine whether NLRP3 inflammasome activation involves Ca^2^⁺ leakage via IP3R, we treated PAMs with 2-APB (an IP3R inhibitor) and dantrolene sodium (a RyR inhibitor). Notably, 2-APB significantly inhibited PRRSV-induced GSDMD cleavage, LDH release, and IL-1β secretion, whereas dantrolene sodium had no effect (Figures 7J–L). In Marc-145 cells overexpressing NSP5, 2-APB efficiently inhibited NSP5-induced increases in cytosolic Ca^2+^ flux, while dantrolene sodium did not (Figure 7M). Confocal microscopy further revealed co-localization of NSP5 with IP3R, supporting the notion that NSP5 causes Ca^2+^ leakage via IP3R channels (Figure 7N). To assess whether 2-APB inhibits NSP5-induced NLRP3 inflammasome activation, we treated Marc-145 cells overexpressing NSP5 with 2-APB or dantrolene sodium. As expected, 2-APB significantly suppressed NSP5-induced GSDMD cleavage and LDH release (Figures 7O–P). Taken together, these findings demonstrate that both intracellular and extracellular Ca^2+^ contribute to PRRSV-induced NLRP3 inflammasome activation. Specifically, NSP5 facilitates Ca^2+^ leakage from the ER into the cytosol via IP3R channels, thereby activating the NLRP3 inflammasome.

### Identification of critical residues in NSP5 for NLRP3 inflammasome activation

**Figure 8:**
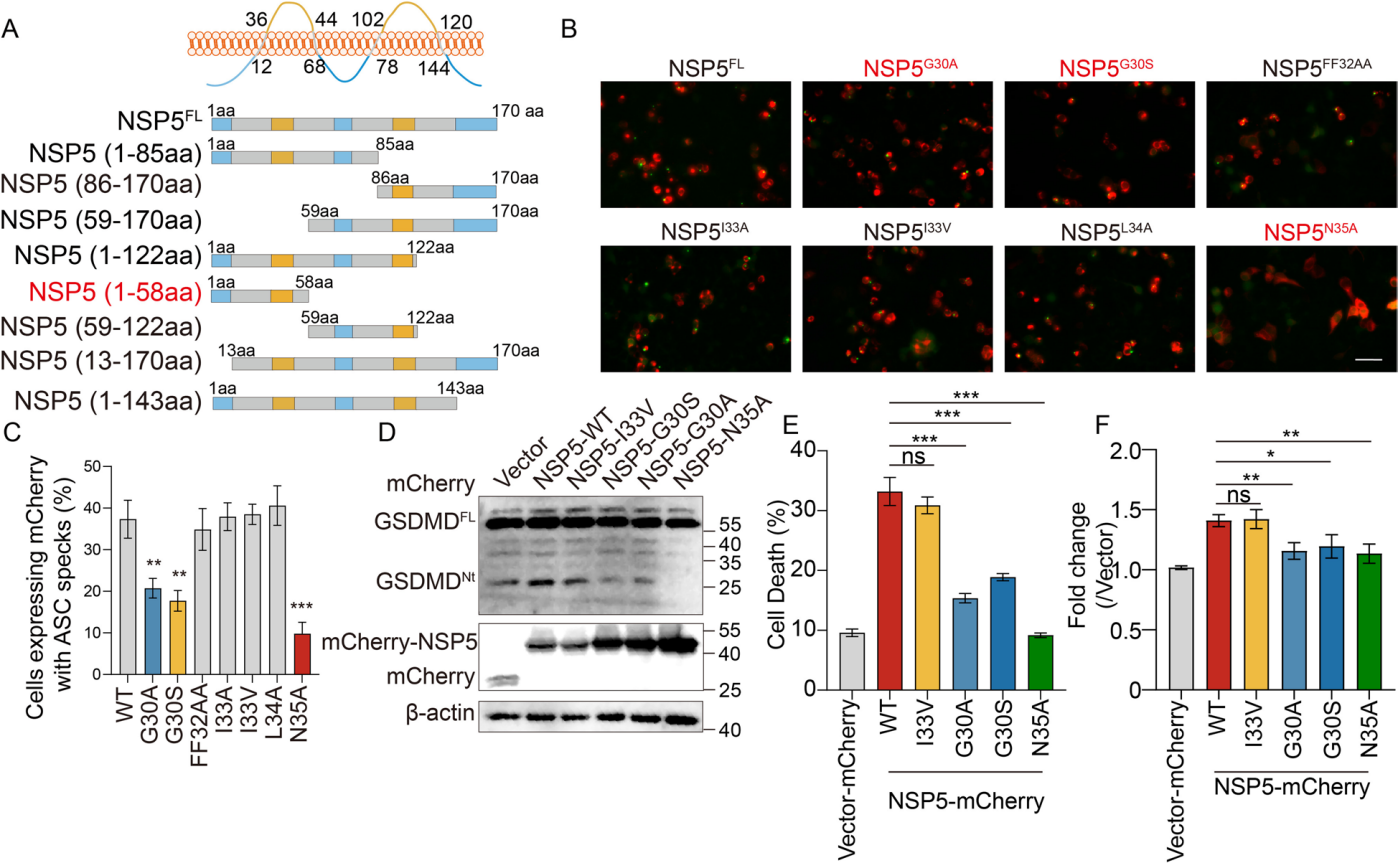
**Identification of critical residues of NSP5 for activating the NLRP3 inflammasome.** **A** Schematic representation of the full-length NSP5 and its deletion mutants. **B** and **C** HEK-293T cells were transfected with a plasmid encoding mCherry-NSP5 or its mutants (G30A, G30S, FF32AA, I33A, I33V, L34A, N35A) in the presence of sNLRP3 inflammasome system (50 ng pCAGGS-sNLRP3-HA, 5 ng pEGFP-C1-ASC, 3 ng pCAGGS-Caspase-1-HA, 200 ng pcDNA3.1-Pro-IL-1β-HA), ASC specks were observed using fluorescence microscopy. ASC specks were visualized using fluorescence microscopy (**B**) and quantified (**C**). Scale bars = 100 μm. The experimental data are representative of results from three independent experiments. **D**–**F** Marc-145 cells were transfected with a plasmid encoding mCherry-NSP5 or its mutants (I33V, G30S, G30A, N35A). Cell lysates were analyzed by western blotting for GSDMD, PRRSV N, and β-actin (**D**). LDH-release-based cell death measurement in the cell culture supernatants was detected by the LDH assay kit (**E**). Cytoplasmic Ca^2+^ was determined by fluorescence of Fluo-8 (**F**). The *p* value of less than 0.05 was considered statistically significant. * for *p* < 0.05, ** for *p* < 0.01, *** for *p* < 0.001, ns for not significant.

To identify the key residues in NSP5 responsible for activating the NLRP3 inflammasome, we constructed eight NSP5 truncations (Figure 8A). Since ER localization of NSP5 is essential for inducing ER stress and Ca^2+^ leakage, we first evaluated whether these truncations retained their ER localization. Transient expression in Marc-145 cells, followed by confocal microscopy, confirmed that all truncations localized to the ER (Additional file 4). Next, we performed a preliminary screening of these truncations using the GFP-ASC speck assay. Co-transfection of NSP5 truncations with NLRP3 and GFP-ASC into HEK-293 T cells revealed that 1-58aa, 1-122aa, 13-170aa, 1-143aa, and 13-58aa truncations were sufficient to induce ASC speck formation, comparable to full-length NSP5 (Additional files 5A and 5B). This finding indicates that the 13-58aa region is critical for NSP5-mediated NLRP3 activation.

To pinpoint key residues within the 13-58aa region, we conducted alanine scanning and generated 14 NSP5 mutants. Co-transfection of these mutants with NLRP3 and GFP-ASC into HEK-293 T cells showed that NSP5 mutants (GFF32AAA and ILN35AAA) targeting residues 30–32 (GFF) and 33–35 (ILN) failed to induce ASC speck formation (Additional files 5C and 5D). To further validate their function, we measured cytoplasmic Ca^2+^ flux in Marc-145 cells transiently expressing GFF32AAA, ILN35AAA, FVL54AAA mutants and wild-type (WT) NSP5. Consistent with the ASC speck results, ILN35AAA and FVL54AAA failed to induce Ca^2+^ release (Additional file 5E).

Sequence alignment of NSP5 from representative PRRSV strains revealed two mutations, G30S and I33V, predominantly found in low-virulence PRRSV strains. To explore their functional significance, we generated substitution mutants (G30A, G30S, FF32AA, I33A, I33V, L34A, N35A) and found that mutations at G30 and N35 significantly reduced ASC speck formation, suggesting that these residues may be critical for NLRP3 activation (Figures 8B and C). To validate these results, we transiently expressed the mutants (G30A, G30S, I33V, and N35A) with wild-type (WT) NSP5 in Marc-145 cells and iPAMs. Our findings revealed that, consistent with WT NSP5, the I33V mutant induced GSDMD cleavage and LDH release (Figures 8D and E and Additional files 5F-G). In contrast, the G30A, G30S, and N35A mutants suppressed GSDMD cleavage and LDH release (Figures 8D and E and Additional files 5F–G). To determine whether these mutations impair NLRP3 activation by affecting Ca^2^⁺ leakage, we transiently expressed the mutants (G30A, G30S, I33V, N35A) and WT NSP5 in Marc-145 cells and measured cytosolic Ca^2^⁺ flux. Consistent with previous findings, the I33V mutant, similar to WT NSP5, induced significant Ca^2^⁺ leakage into the cytosol, whereas the G30A, G30S, and N35A mutants exhibited minimal effects on Ca^2^⁺ leakage (Figure 8F). These results underscore the pivotal roles of G30 and N35 in NSP5-mediated NLRP3 activation and downstream signaling.

### The PRRSV mutant carrying the NSP5 G30A mutation significantly attenuates NLRP3 inflammasome activation

**Figure 9:**
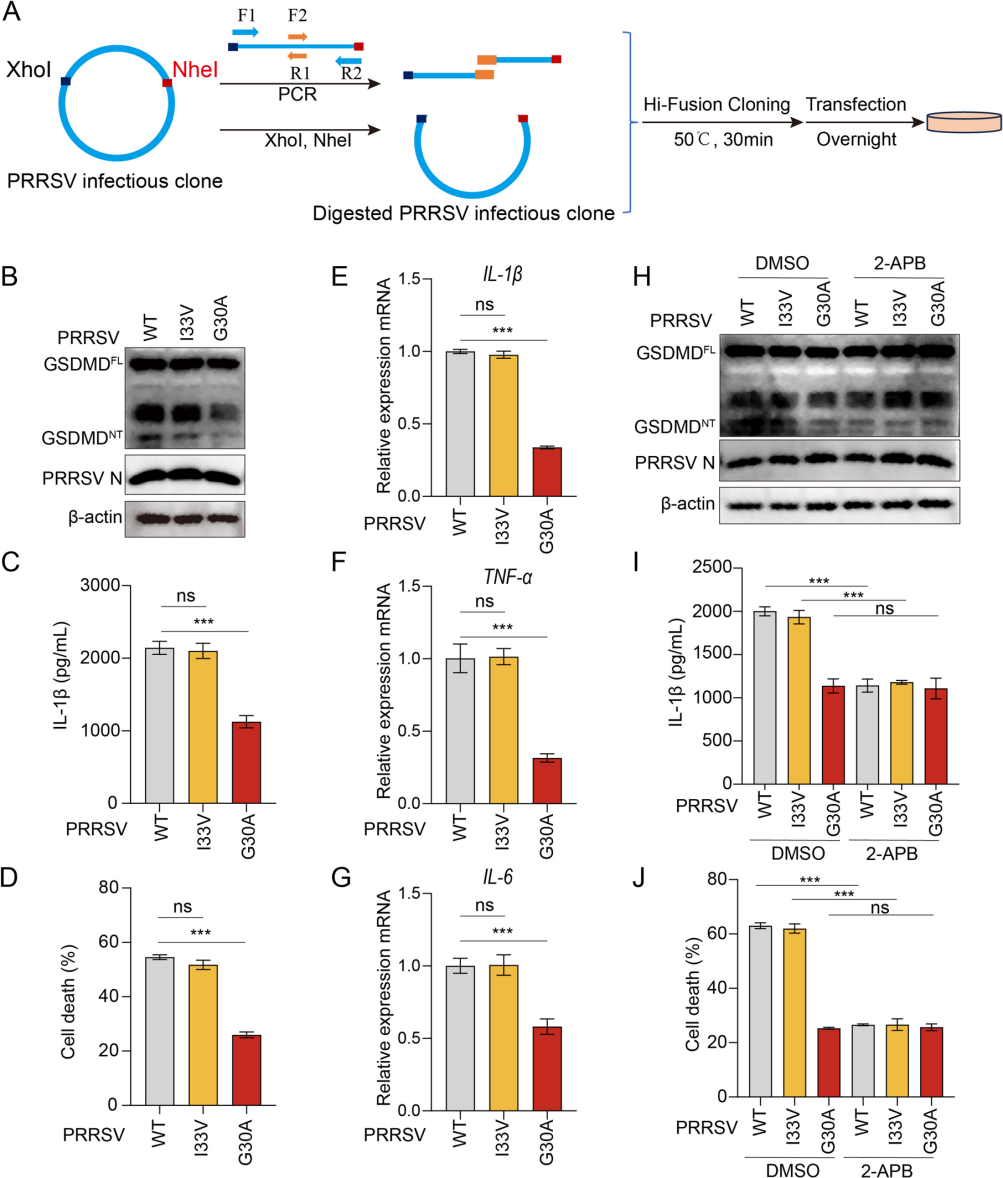
**The PRRSV mutant virus carrying the NSP5 G30A mutation significantly attenuates NLRP3 inflammasome activation.** **A** Schematic diagram of rescuing PRRSV mutant viruses. **B**–**D** PAMs were infected with the indicated viruses at an MOI of 1 for 24 h. Cell lysates were analyzed by western blotting for GSDMD, PRRSV N, and β-actin (**B**). Swine IL-1β levels in the cell culture supernatants were detected by ELISA (**C**). LDH-release-based cell death measurement in the cell culture supernatants was detected by the LDH assay kit (**D**). **E**–**G** Quantitative analysis of the relative mRNA abundance of IL-1β (**E**), TNF-α (**F**), and IL-6 (**G**) via qPCR that was normalized against GAPDH and compared to the WT-infected. **H** and **J** PAMs were infected with PRRSV (MOI = 1) and treated with DMSO and 2-APB for 24 h. Cell lysates were analyzed by western blotting for GSDMD, PRRSV N, and β-actin (**H**). Swine IL-1β levels in the cell culture supernatants were detected by ELISA (**I**). LDH-release-based cell death measurement in the cell culture supernatants was detected by the LDH assay kit (**J**). The *p* value of less than 0.05 was considered statistically significant. *** for *p* < 0.001, ns for not significant.

To investigate the role of specific NSP5 residues in NLRP3 inflammasome activation, we introduced targeted point mutations into the infectious cDNA clone of PRRSV in a DNA-launched system by site-directed mutagenesis. Three NSP5 mutants were generated: I33V, G30A, and N35A (Figure 9A). The cDNA plasmids were then transfected into Marc-145 cells to rescue live viruses. Out of the three mutants, only two mutants (I33V and G30A) were successfully rescued, while N35A could not be recovered (Additional files 6A-6C). To evaluate the impact of the G30A mutation on NLRP3 inflammasome activation, primary PAMs were infected with the WT, I33V, or G30A viruses. Compared to WT and I33V viruses, infection with G30A virus resulted in significantly lower levels of GSDMD cleavage, LDH release, and IL-1β secretion (Figures 9B–D). Additionally, RT-qPCR analysis demonstrated that G30A-infected primary PAMs exhibited markedly reduced mRNA levels of inflammatory cytokines, including *IL-1β*, *TNF-α*, and *IL-6*, compared to WT and I33V mutant viruses (Figures 9E–G).

Since our previous study revealed that NSP5 triggers Ca^2+^ leakage via IP3R to activate the NLRP3 inflammasome, we examined whether the G30A mutation disrupts this process. Primary PAMs were pretreated with the IP3R-specific inhibitor 2-APB before infection. The results showed that WT and I33V infections led to significant GSDMD cleavage, LDH release, and IL-1β secretion, all of which were markedly reduced upon 2-APB treatment. However, in cells infected with G30A, these inflammatory responses were minimally affected by 2-APB (Figures 9H–J), indicating the G30A mutation abolished PRRSV’s ability to induce Ca^2+^ leakage via IP3R. Collectively, these findings demonstrate that PRRSV mutant virus carrying the NSP5 G30A mutation significantly attenuates the NLRP3 inflammasome activation.

### NSP5 inhibits IFN-I pathway by blocking STING trafficking

**Figure 10:**
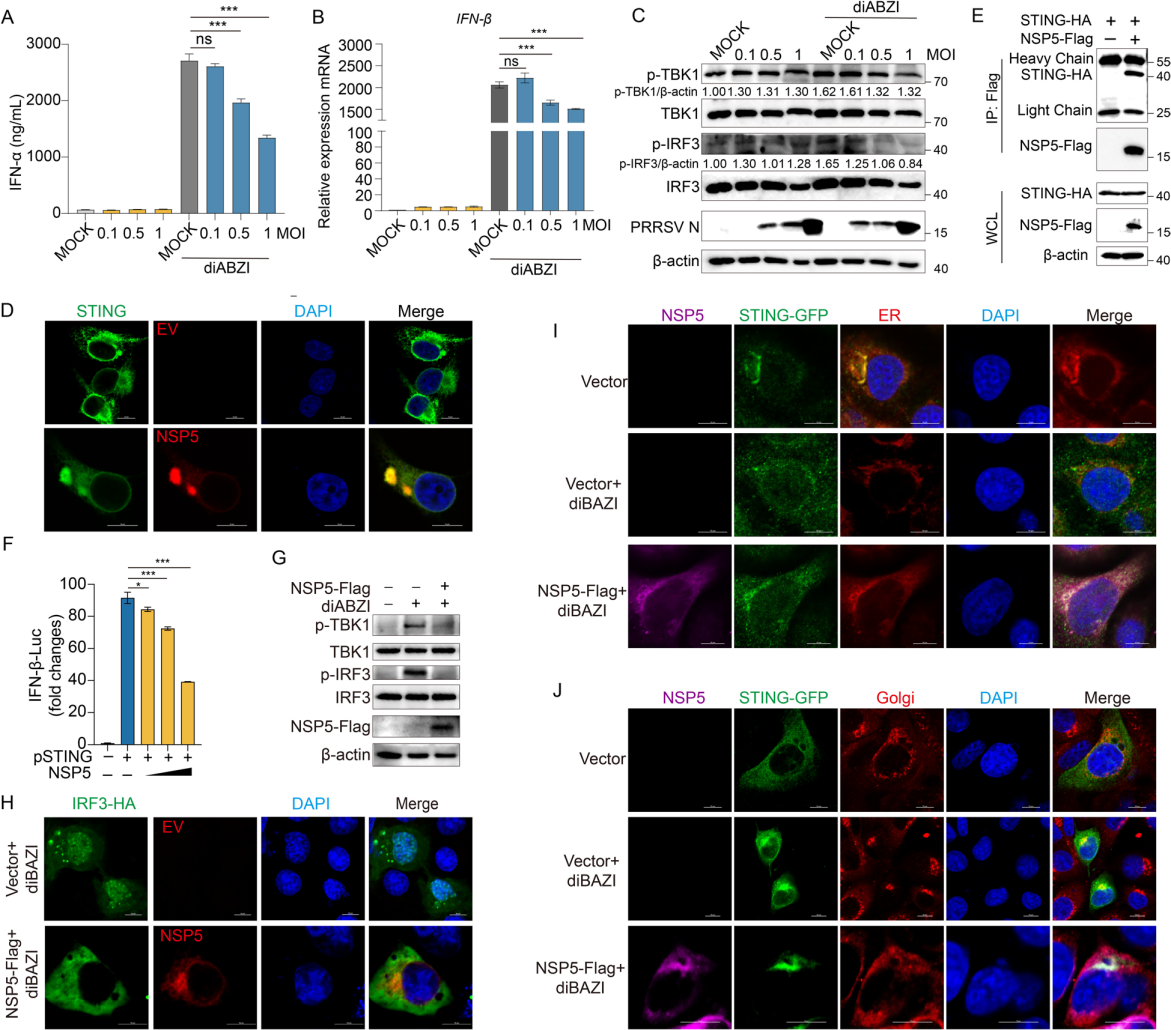
**NSP5 inhibits IFN-I pathway by blocking STING trafficking.** **A**–**C** PAMs were infected with PRRSV at various multiplicities of infection (MOIs) for 24 h, the cells were treated with diABZI at a concentration of 15 μM for 12 h before being collected. Swine IFN-α levels in the cell culture supernatants were detected by ELISA (**A**). Swine *IFN-β* mRNA levels (related to swine GAPDH) were analyzed by quantitative RT-PCR (qPCR) (**B**). Immunoblot analysis of TBK1, IRF3, and their phosphorylated forms. The relative band density of p-TBK1 and p-IRF3 was normalized to β-actin (**C**). **D** Co-localization of NSP5 with STING was analyzed in Marc-145 cells transfected with NSP5-Flag using confocal microscopy. Scale bars = 10 μm. **E** HEK-293T cells were co-transfected with a plasmid encoding NSP5-Flag and a plasmid encoding STING-HA. **F** Detection of IFN-β promoter activation in HEK-293 T cells transfected with IFN-β-Luc reporter, expression plasmids encoding pSTING and PRRSV NSP5 or empty vector for 24 h using dual-luciferase reporter assay. **G** Marc-145 cells were transfected with a plasmid encoding NSP5-Flag for 24 h with or without diABZI. **H** PRRSV NSP5 inhibits IRF3 translocated into the nucleus. Marc-145 cells were co-transfected with pcDNA3.1-NSP5-Flag or pcDNA3.1 and IRF3-HA for 24 h with or without diABZI. Scale bars = 10 μm. I and J PRRSV NSP5 blocks STING trafficking. Marc-145 cells were co-transfected with pcDNA3.1-NSP5-Flag or pcDNA3.1 and STING-GFP for 24 h with or without diABZI. **I** The cells were incubated with anti-Flag (pink) and anti-GORASP2 (Golgi marker, red) and stained with DAPI (blue). **J** The cells were incubated with anti-Flag (pink) and stained with ER-specific fluorescent dye (red) and DAPI (blue). Scale bars = 10 μm. The *p* value of less than 0.05 was considered statistically significant. * for *p* < 0.05, ** for *p* < 0.01, *** for *p* < 0.001, ns for not significant.

Previous studies have demonstrated that PRRSV infection can trigger the release of mitochondrial DNA (mtDNA) and activate the cytoplasmic cGAS-STING pathway [65]. However, our ELISA results showed no significant increase in IFN-α secretion following PRRSV infection. Given the low levels of IFN-α, we treated PAMs with the STING agonist diABZI and found that PRRSV infection significantly inhibited IFN-α secretion in a dose-dependent manner (Figure 10A). Similarly, after stimulation with the STING agonist diABZI, PRRSV infection markedly suppressed the mRNA levels of *IFN-β* and *ISGs* (*ISG15*, *ISG54*, *ISG56*, *Mx1*, *Mx2* and *OAS1b*) (Figure 10B and Additional files 7A-7F). To further investigate the effect of PRRSV on STING activation, we examined the phosphorylation of TBK1 and IRF3, two key downstream indicators of STING pathway activation. Even under diABZI stimulation, PRRSV infection significantly inhibited the phosphorylation of TBK1 and IRF3 in a dose-dependent manner (Figure 10C). These findings collectively demonstrate that PRRSV infection triggers pyroptosis and excessive inflammatory responses while simultaneously suppressing STING signaling and interferon production, leading to the observed phenomenon of “excessive inflammation but insufficient antiviral response”. In the IFN-I signaling pathway, STING, a critical immune adaptor protein, is localized in the ER, as is NSP5. This prompted us to hypothesize a potential interaction between NSP5 and STING. Using confocal microscopy, we observed co-localization of endogenous STING with NSP5 (Figure 10D). Additionally, Co-IP assays confirmed an interaction between STING and NSP5 (Figure 10E). To investigate whether NSP5 suppresses STING activation, we performed a dual-luciferase reporter assay. The results showed that NSP5 overexpression inhibited STING-induced IFN-β promoter activation in a dose-dependent manner (Figure 10F). To further confirm that NSP5 disrupts STING signaling, we stimulated Marc-145 cells and iPAMs with diABZI, a STING agonist. In control cells, diABZI treatment significantly increased the phosphorylation of TBK1 and IRF3. However, in NSP5-overexpressing cells, even with diABZI stimulation, phosphorylation levels of TBK1 and IRF3 were significantly reduced compared to the diABZI-treated control group (Figure 10G and Additional file 8A).

Nuclear translocation of IRF3 is a hallmark of innate immune activation [66, 67]. We found that diABZI stimulation led to IRF3 translocation into the nucleus, forming distinct clusters. However, in NSP5-overexpressing cells, even with diABZI stimulation, IRF3 remained retained in the cytoplasm, failing to translocate to the nucleus (Figure 10H), indicating that NSP5 inhibits STING signaling. Since both NSP5 and STING localize to the ER, we hypothesized that NSP5 might impair STING trafficking. Under normal condition, upon activation, STING translocates from the ER to the Golgi [17], where it interacts with TBK1 and IRF3, leading to IRF3 activation. However, in NSP5-overexpressing cells, even after diABZI stimulation, STING remained trapped in the ER and failed to translocate to the Golgi (Figure 10I and Additional file 8B). Confocal microscopy further confirmed that in diABZI-stimulated control cells, STING prominently localized to the Golgi. In contrast, in NSP5-overexpressing cells, STING failed to reach the Golgi (Figure 10J). Taken together, these findings demonstrate that PRRSV NSP5 prevents STING trafficking from the ER to the Golgi, thereby suppressing STING-mediated activation of IFN-I pathway.

## Discussion

**Figure 11:**
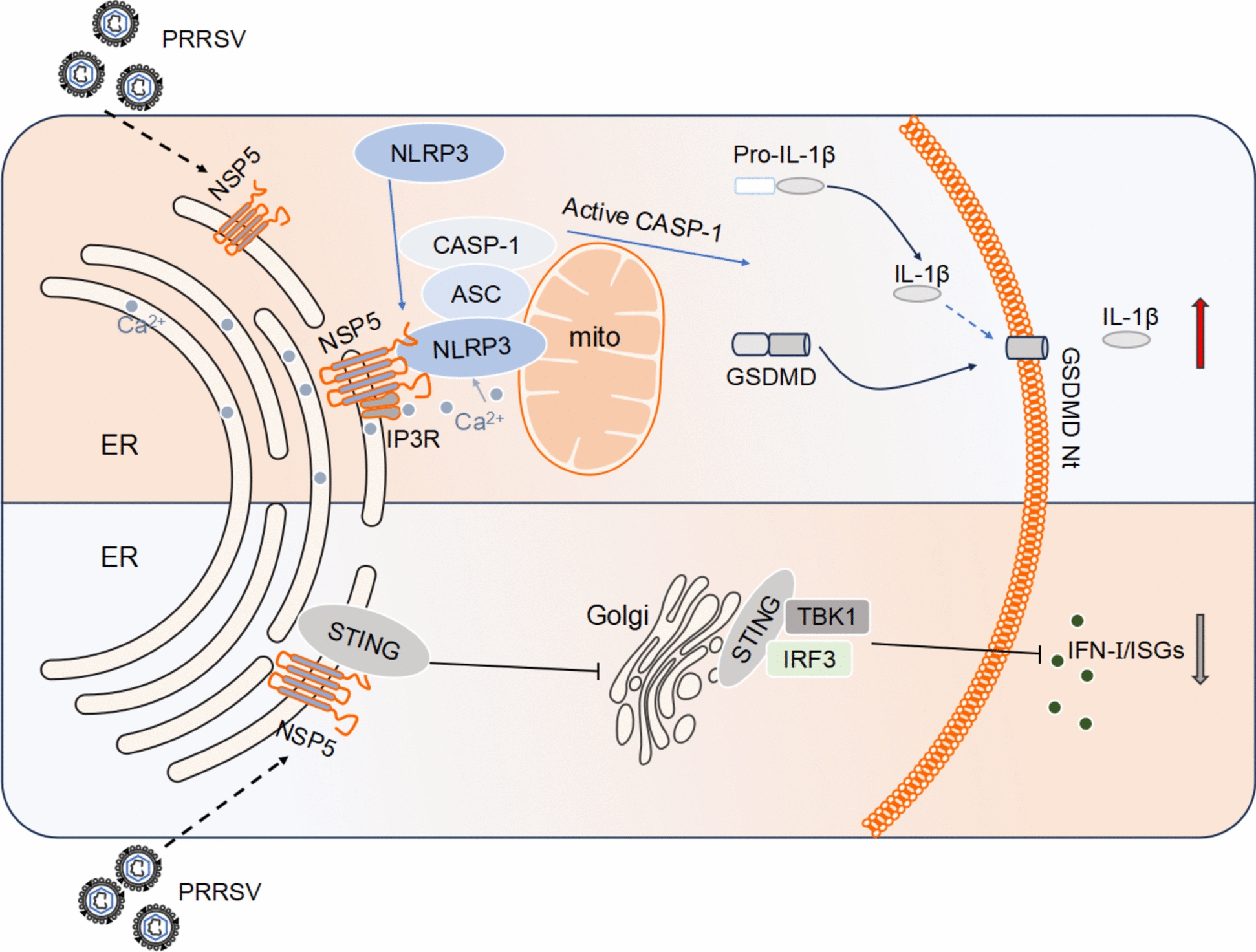
**Proposed model of PRRSV NSP5-mediated NLRP3 inflammation activation and IFN-I suppression.** NSP5 interacts with NLRP3, recruiting it to the ER-mitochondria (mito) contact sites. Simultaneously, NSP5 induces Ca^2^⁺ leakage from the ER, which is sensed by NLRP3 at these contact sites, leading to NLRP3 inflammasome assembly and activation. Additionally, NSP5 interacts with STING, preventing its translocation to the Golgi apparatus, thereby suppressing productions of IFN-I and ISGs. This dual mechanism ultimately results in an imbalanced immune response characterized by excessive inflammation and reduced IFN-I production during PRRSV infection. GSDMD Nt, N-terminal domain of GSDMD.

The inflammasome and IFN-I pathways are two critical branches of innate immunity that play essential roles in defending against viral infections. However, an imbalance between these two pathways has emerged as a key viral immune evasion strategy [32, 33]. Previous studies have shown that vaccinia virus (VACV) and Sendai virus (SeV) activate inflammasomes, leading to Caspase-1 activation, which subsequently cleaves cGAS to suppress IFN-I production [29]. However, no prior report has identified a single viral protein that simultaneously activates inflammasomes while suppressing IFN-I induction. In this study, we elucidate the molecular mechanism by which PRRSV infection induces high levels of inflammation and low levels of interferon. Our findings demonstrate that the PRRSV non-structural protein NSP5 localizes in the ER and interacts with NLRP3, recruiting it to the ER-mitochondria interface. Meanwhile, NSP5 induces ER stress, leading to ER Ca^2^⁺ leakage, which in turn activates the NLRP3 inflammasome, triggering inflammation. Moreover, NSP5 interacts with STING, preventing its translocation from the ER to the Golgi apparatus, thereby blocking STING signaling and ultimately suppressing IFN-I (Figure 11).

Previous studies have demonstrated that NLRP3 translocation to the ER-mitochondria interface is a critical step for its activation during viral infections [54]. Herpes simplex virus type 1 (HSV-1) recruits NLRP3 to the ER via STING, facilitating inflammasome assembly [68], while SeV utilizes MAVS to recruit NLRP3 to mitochondria, promoting its oligomerization [69]. However, whether PRRSV infection triggers similar translocation was previously unclear. Our data demonstrate that NLRP3 undergoes translocation to the ER and mitochondria during PRRSV infection. Further analysis shows that the PRRSV NSP5 interacts with the NACHT domain of NLRP3, recruiting it to the ER-mitochondria interface, where it facilitates NLRP3 inflammasome activation.

NLRP3 activation requires specific signals, with Ca^2^⁺ being a well-established activation signal [8, 9]. Previous studies have reported that the 2B proteins of picornaviruses, including EMCV, rhinovirus, and foot-and-mouth disease virus, localize to the ER and mediate Ca^2^⁺ leakage into the cytoplasm via their ion channel activity, leading to the NLRP3 activation [70–72]. Similarly, the E protein of SARS-CoV-2 forms protein-lipid channels to induce Ca^2^⁺ leakage, activating NLRP3 [73], while the core protein of HCV induces Ca^2^⁺ leakage through phospholipase C (PLC), also leading to NLRP3 activation [74]. Our findings indicate that PRRSV NSP5 primarily localizes to the ER and induces significant ER stress. Given the close link between Ca^2^⁺ homeostasis and ER stress, we hypothesized that NSP5 might cause ER Ca^2^⁺ leakage, triggering NLRP3 inflammasome activation. Indeed, we observed that NSP5-expressing cells exhibited significantly increased cytosolic Ca^2^⁺ flux, confirming that NSP5 functions as a Ca^2^⁺-modulating factor in PRRSV-induced NLRP3 inflammasome activation. Further experiments demonstrate that both intracellular and extracellular Ca^2^⁺ are required for PRRSV-induced NLRP3 inflammasome activation. Our study shows that NSP5 mediates Ca^2^⁺ leakage through IP3R ion channels rather than RyRs ion channels, ultimately resulting in NLRP3 activation. Although previous studies have shown that PRRSV GP5 induces mtROS production to activate both autophagy and NLRP3 inflammasome, autophagy also mitigates NLRP3-mediated inflammation, thereby promoting viral replication [51]. However, GP5 was not the dominant protein activating the NLRP3 inflammasome in our screening system. Additionally, NSP2 has been reported to enhance the interaction between NLRP3 and IKKβ, driving NLRP3 translocation to the trans-Golgi network sites for oligomerization. However, these studies were mainly conducted in THP-1 cells, a non-PRRSV-susceptible cell line [75]. Moreover, prior research has also indicated that NSP5 is the most potent protein in activating the NLRP3 inflammasome [76]. To identify the key residues responsible for NSP5-mediated NLRP3 activation, we performed NSP5 truncation and mutational analysis, followed by ASC-speck screening assay. The results identified G30 and N35 as critical residues for NLRP3 activation. Using a reverse genetics system, we successfully generated G30A and I33V mutant viruses, but N35-targeted mutants were not viable, suggesting that N35 is essential for viral survival. Notably, the G30A mutant virus exhibited significantly lower LDH release and IL-1β secretion compared to the WT and I33V mutant viruses, confirming that G30 is a critical residue for NSP5-mediated NLRP3 activation.

Although the cGAS-STING pathway is primarily recognized for sensing DNA viruses, increasing evidence suggests its involvement in RNA virus defense [22–25]. STING agonists have been shown to inhibit SARS-CoV-2 replication by activating STING-mediated antiviral immunity, whereas SARS-CoV-2 ORF10 antagonizes STING-mediated antiviral responses. Similarly, flavivirus NS2B3 proteases, such as those from dengue virus and Zika virus, cleave STING to suppress IFN-I production [22, 23].

Previous studies have reported that PRRSV infection triggers mtDNA release, which activates the cGAS-STING pathway [65]. However, our findings demonstrate that PRRSV infection induces minimal IFN-I production. Furthermore, stimulation with the STING agonist following PRRSV infection revealed that PRRSV significantly suppresses STING-activated IFN-I induction, suggesting that PRRSV inhibits cGAS-STING signaling to counteract the IFN-I pathway. In this study, we identified a direct interaction between PRRSV NSP5 and STING, with NSP5 preventing STING translocation from the ER to the Golgi, thereby blocking STING activation. Using confocal microscopy, we observed that, even under STING agonist diABZI stimulation, the presence of NSP5 caused STING retention in the ER, preventing its translocation to the Golgi apparatus. These findings highlight NSP5 as a key PRRSV’s antagonist of the IFN-I pathway, disrupting STING-mediated antiviral signaling.

In conclusion, our study identifies a unique viral protein that plays a dual role in activating the NLRP3 inflammasome while simultaneously suppressing STING trafficking to attenuate IFN-I production. This dual function provides novel insights into how the virus mediates host immune dysregulation through a single protein, shedding light on a previously unrecognized immune evasion strategy. Moreover, this discovery presents a promising target for the development of vaccines and antiviral therapies against PRRSV and may inspire further investigations into similar mechanisms in other viruses, paving the way for broader advancements in understanding immune evasion.

## Supplementary Information

**Additional file 1.** **Quantification of PRRSV N, *IL-6*** **and** ***TNF-α*** **mRNA levels following PRRSV infection.** PAMs were infected with PRRSV at different MOIs or at MOI = 1 for various time points. (A and B) The relative mRNA abundance of PRRSV *N* was measured via qPCR, normalized against GAPDH, and compared to the uninfected MOCK group. (C to F) The relative mRNA abundance of *IL-6* and *TNF-α* was measured via qPCR, normalized against GAPDH, and compared to the uninfected MOCK group. The *p* value of less than 0.05 was considered statistically significant. *** for *p* < 0.001.

**Additional file 2.** **ASC oligomerization during PRRSV infection and the effect of NSP5 overexpression on ASC speck formation.** (A) Marc-145 cells were infected with PRRSV at an MOI of 1, and samples were collected at various time points. The cell lysates were prepared, and the pellets were washed with PBS for three times and cross-linked using DSS for western blotting. (B) iPAMs were transfected with plasmids encoding sNLRP3-HA, sASC-HA and NSP5-Flag or NSP11-Flag, followed by LPS treatment. iPAMs were treated with LPS for 8 h followed by nigericin for 4 h as positive controls. At 36 hpt, the cells were then fixed and probed with anti-ASC (green) and anti-Flag (red) antibodies, and nucleus marker DAPI (blue), and then observed by confocal microscopy. Scale bars = 10 μm.

**Additional file 3.** **Effect of NSP5 on NLRP3 mRNA levels and its subcellular localization analysis.** (A and B) Marc-145 cells and iPAMs were transfected with different doses of NSP5-Flag, and samples were collected at 36 hpt for qPCR analysis. (C, E and G) Co-localization of NSP5 with the ER, Mitochondria or Golgi was analyzed using confocal microscopy. Scale bars = 10 μm. (D, F and H) The colocalization was analyzed by ImageJ software. Images are representative of 3 biological replicates.

**Additional file 4. Co-localization of NSP5 truncation mutants with the ER.** Marc-145 cells were co-transfected with a plasmid encoding RR-mNeonGreen (ER marker) and mCherry-NSP5 or its deletion mutants (1-85aa, 86-170aa, 59-170aa, 1-122aa, 1-58aa, 59-122aa, 13-170aa, 1-143aa), and then observed by confocal microscopy. Scale bars = 10 μm. The experimental data are representative of results from three independent experiments.

**Additional file 5.** **Identification of key residues essential for NSP5-induced NLRP3 activation. (**A and B) HEK-293T cells were transfected with a plasmid encoding mCherry-NSP5 or its deletion mutants (1-85aa, 86-170aa, 59-170aa, 1-122aa, 1-58aa, 59-122aa, 13-170aa, 1-143aa) in the presence of sNLRP3 inflammasome system (50 ng pCAGGS-sNLRP3-HA, 5 ng pEGFP-C1-ASC, 3 ng pCAGGS-Caspase-1-HA, 200 ng pcDNA3.1-Pro-IL-1β-HA). ASC specks were visualized by fluorescence microscopy (A) and quantified (B). Scale bars = 100 μm. (C and D) HEK-293 T cells were transfected with a plasmid encoding mCherry-NSP5 or its mutants (fourteen mutants) in the presence of sNLRP3 inflammasome system (50 ng pCAGGS-sNLRP3-HA, 5 ng pEGFP-C1-ASC, 3 ng pCAGGS-Caspase-1-HA, 200 ng pcDNA3.1-Pro-IL-1β-HA). ASC specks were visualized by fluorescence microscopy (C) and quantified (D). Scale bars = 100 μm. (E) Marc-145 cells were transfected with a plasmid encoding mCherry-NSP5 or its mutants (GFF32AAA, ILN35AAA, FVL54AAA). Cytoplasmic Ca^2+^ levels were determined by fluorescence of Fluo-8. (F and G) iPAMs were transfected with a plasmid encoding mCherry-NSP5 or its mutants (I33V, G30S, G30A, N35A). Cell lysates were analyzed by western blotting for GSDMD, PRRSV-N, and β-actin (F). LDH-release-based cell death measurement in the cell culture supernatants were detected by LDH assay kit (G). * for *p* < 0.05, ** for *p* < 0.01, *** for *p* < 0.001, ns for not significant. The experimental data are representative of results from three independent experiments.

**Additional file 6.** **Rescue of PRRSV carrying the NSP5 mutations.** (A) Immunofluorescence assay (IFA) of rescued PRRSV. Harvested transfected cells were infected with Marc-145 cells. Cells were stained with anti-M antibodies (Green). Scale bars = 50 μm. (B) DNA sequencing of PRRSV cDNA clones of wild-type, I33V and G30A.

**Additional file 7.** **Quantification of ISG mRNA levels following PRRSV infection.** (A to F) PAMs were infected with PRRSV at different MOIs for 24 h. The relative mRNA abundance of *ISG15*, *ISG54*, *ISG56*, *Mx1*, *Mx2* and *OAS1b* was measured via qPCR, normalized against GAPDH, and compared to the uninfected MOCK group. The *p* value of less than 0.05 was considered statistically significant. * for *p* < 0.05, ** for *p* < 0.01, *** for *p* < 0.001, ns for not significant.

**Additional file 8.** **PRRSV NSP5 blocks STING trafficking.** (A) iPAMs were transfected with a plasmid encoding NSP5-Flag for 24 h with or without diABZI. Cell lysates were analyzed by western blotting for p-TBK1, TBK1, p-IRF3, IRF3, Flag, and β-actin. (B) iPAMs were co-transfected with pcDNA3.1-NSP5-Flag or pcDNA3.1 and pcDNA3.1-STING-HA for 24 h with or without diABZI. The cells were incubated with anti-HA (green) and anti-Flag (pink) and stained with ER-specific fluorescent dye (red) and DAPI (blue) for confocal microscopy analysis. Scale bars = 10 μm.

## Data Availability

All relevant data are included in the manuscript and Additional files.
