# Supplementary figures and images for: PRRSV NSP5 orchestrates dual immune disruption by targeting NLRP3 and STING
Source: Vet Res. 2025 Oct 16;56:199. doi: 10.1186/s13567-025-01636-3 (PMC12533338; doi:10.1186/s13567-025-01636-3)

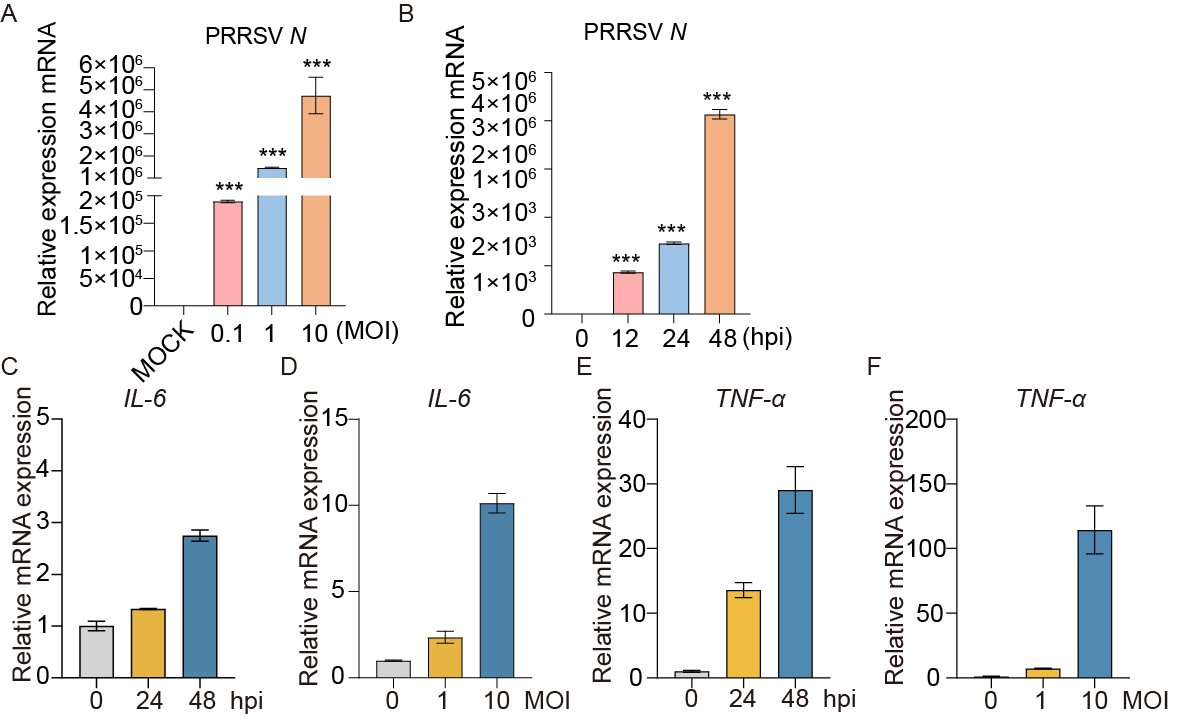

Supplement: Supplementary file 1 — Additional file 1. Quantification of PRRSV N, IL-6 and TNF-α mRNA levels following PRRSV infection. PAMs were infected with PRRSV at different MOIs or at MOI = 1 for various time points. (A and B) The relative mRNA abundance of PRRSV N was measured via qPCR, normalized against GAPDH, and compared to the uninfected MOCK group. (C to F) The relative mRNA abundance of IL-6 and TNF-α was measured via qPCR, normalized against GAPDH, and compared to the uninfected MOCK group. The p value of less than 0.05 was considered statistically significant. *** for p < 0.001. [file 13567_2025_1636_MOESM1_ESM.tif]

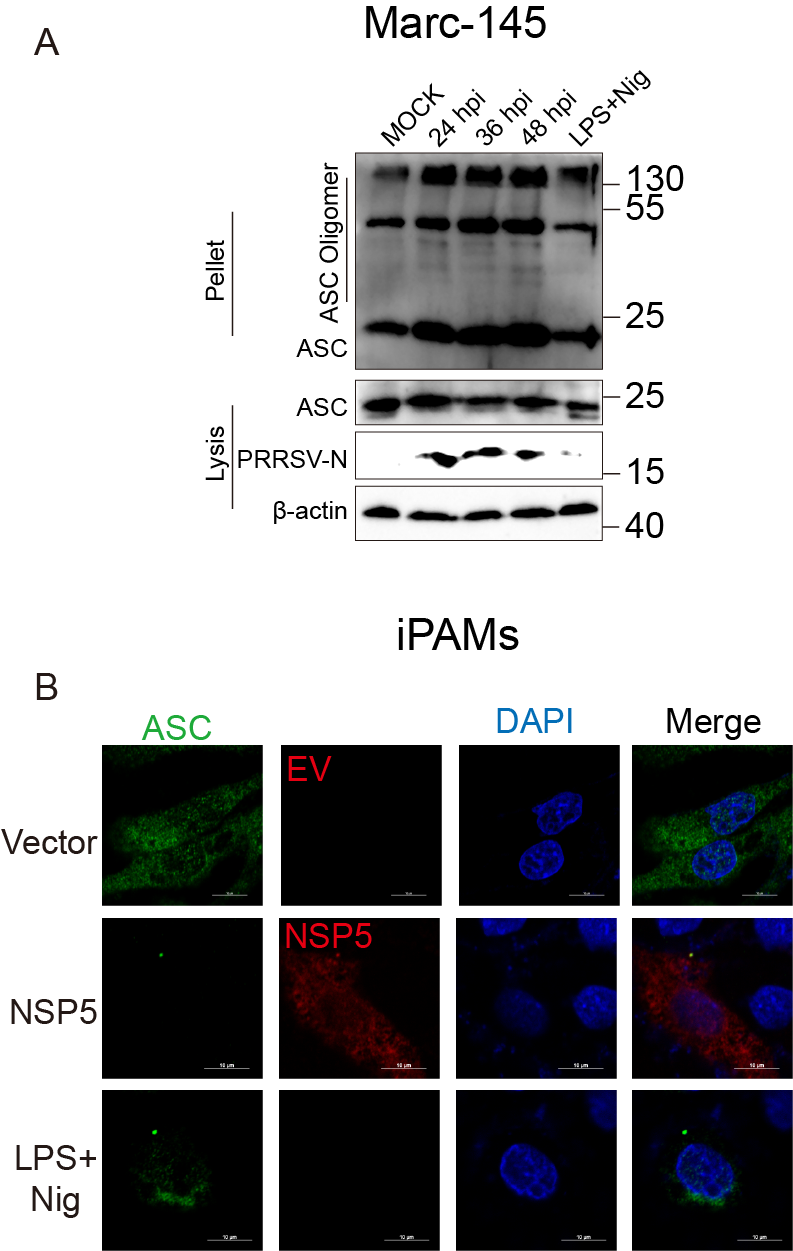

Supplement: Supplementary file 2 — Additional file 2. ASC oligomerization during PRRSV infection and the effect of NSP5 overexpression on ASC speck formation. (A) Marc-145 cells were infected with PRRSV at an MOI of 1, and samples were collected at various time points. The cell lysates were prepared, and the pellets were washed with PBS for three times and cross-linked using DSS for western blotting. (B) iPAMs were transfected with plasmids encoding sNLRP3-HA, sASC-HA and NSP5-Flag or NSP11-Flag, followed by LPS treatment. iPAMs were treated with LPS for 8 h followed by nigericin for 4 h as positive controls. At 36 hpt, the cells were then fixed and probed with anti-ASC (green) and anti-Flag (red) antibodies, and nucleus marker DAPI (blue), and then observed by confocal microscopy. Scale bars = 10 μm. [file 13567_2025_1636_MOESM2_ESM.tif]

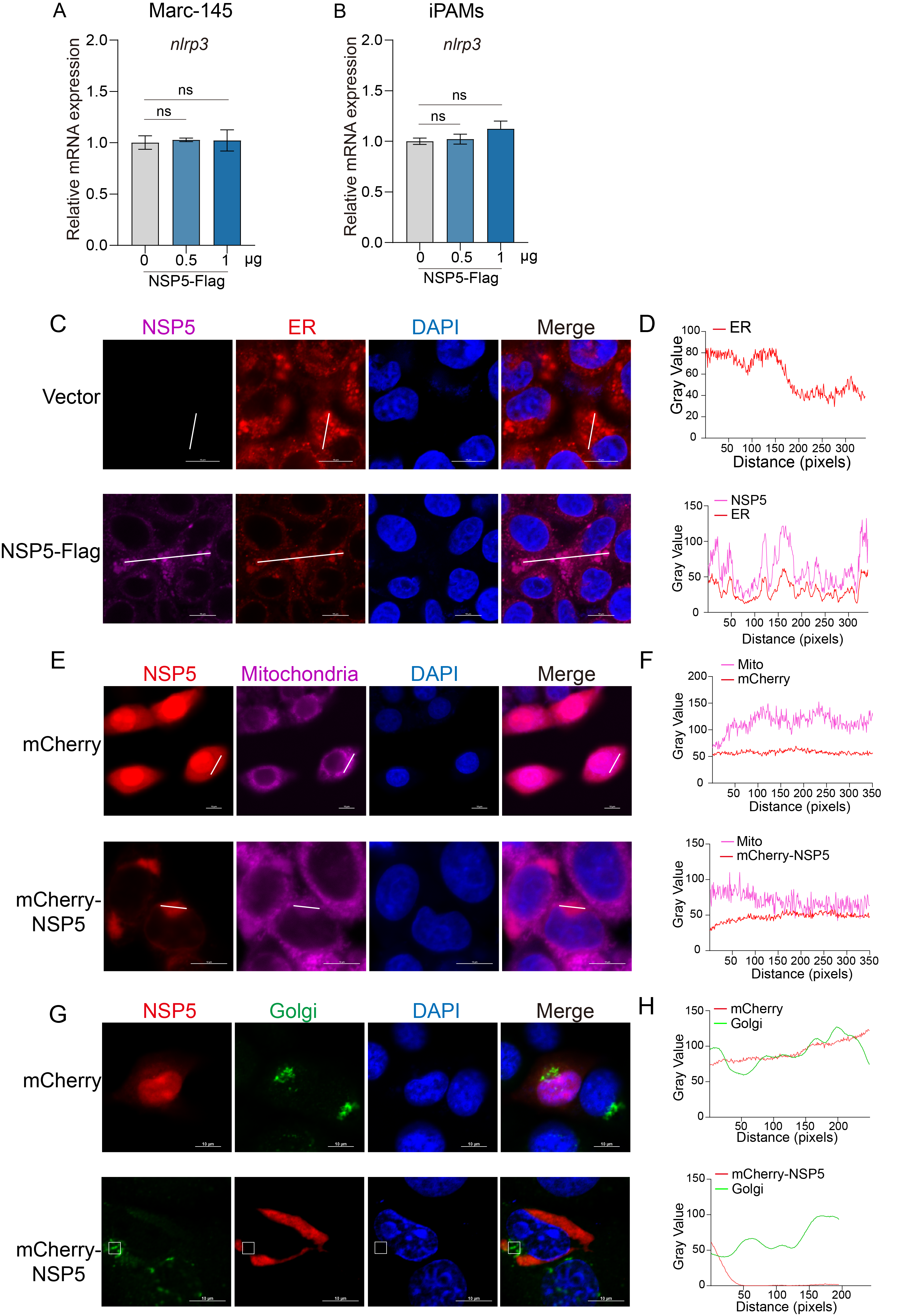

Supplement: Supplementary file 3 — Additional file 3. Effect of NSP5 on NLRP3 mRNA levels and its subcellular localization analysis. (A and B) Marc-145 cells and iPAMs were transfected with different doses of NSP5-Flag, and samples were collected at 36 hpt for qPCR analysis. (C, E and G) Co-localization of NSP5 with the ER, Mitochondria or Golgi was analyzed using confocal microscopy. Scale bars = 10 μm. (D, F and H) The colocalization was analyzed by ImageJ software. Images are representative of 3 biological replicates. [file 13567_2025_1636_MOESM3_ESM.tif]

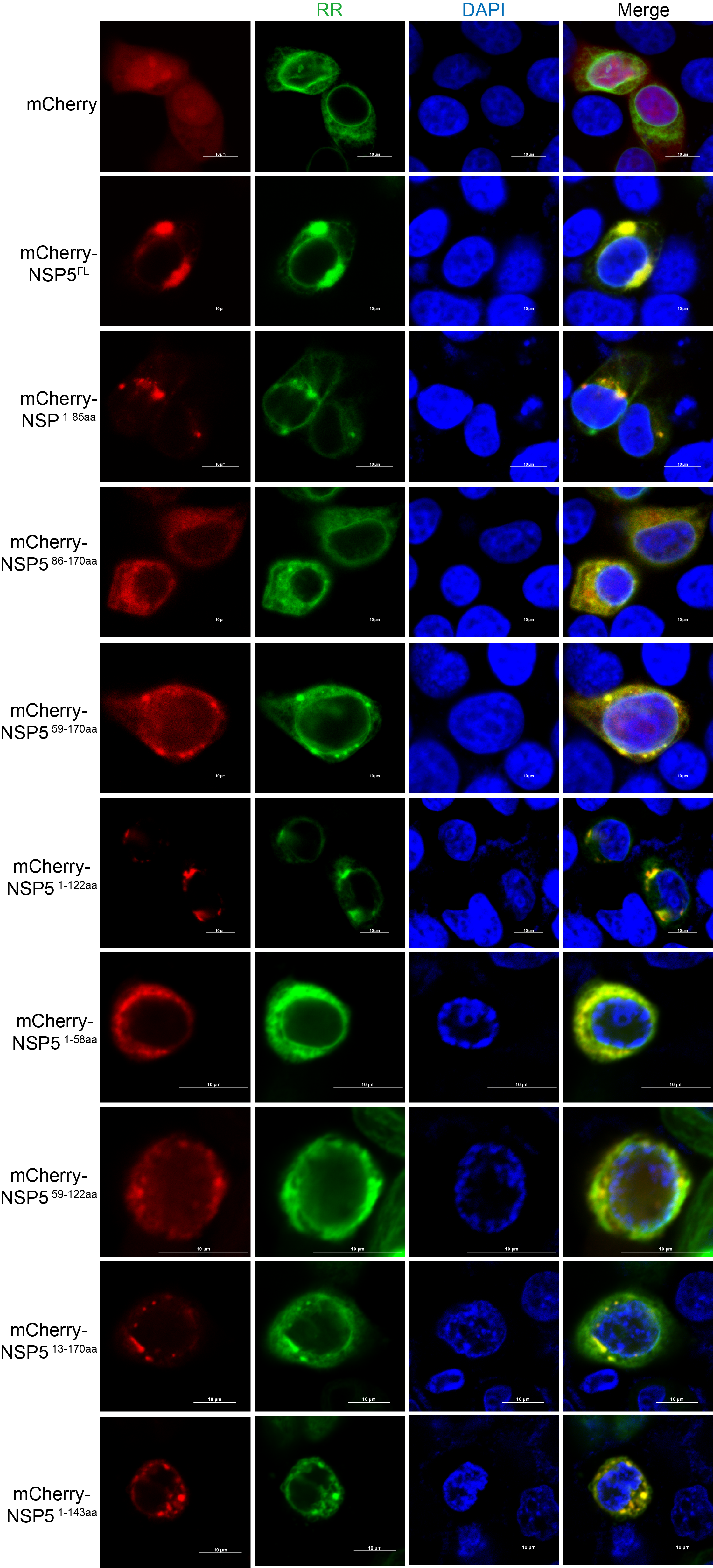

Supplement: Supplementary file 4 — Additional file 4. Co-localization of NSP5 truncation mutants with the ER. Marc-145 cells were co-transfected with a plasmid encoding RR-mNeonGreen (ER marker) and mCherry-NSP5 or its deletion mutants (1-85aa, 86-170aa, 59-170aa, 1-122aa, 1-58aa, 59-122aa, 13-170aa, 1-143aa), and then observed by confocal microscopy. Scale bars = 10 μm. The experimental data are representative of results from three independent experiments. [file 13567_2025_1636_MOESM4_ESM.tif]

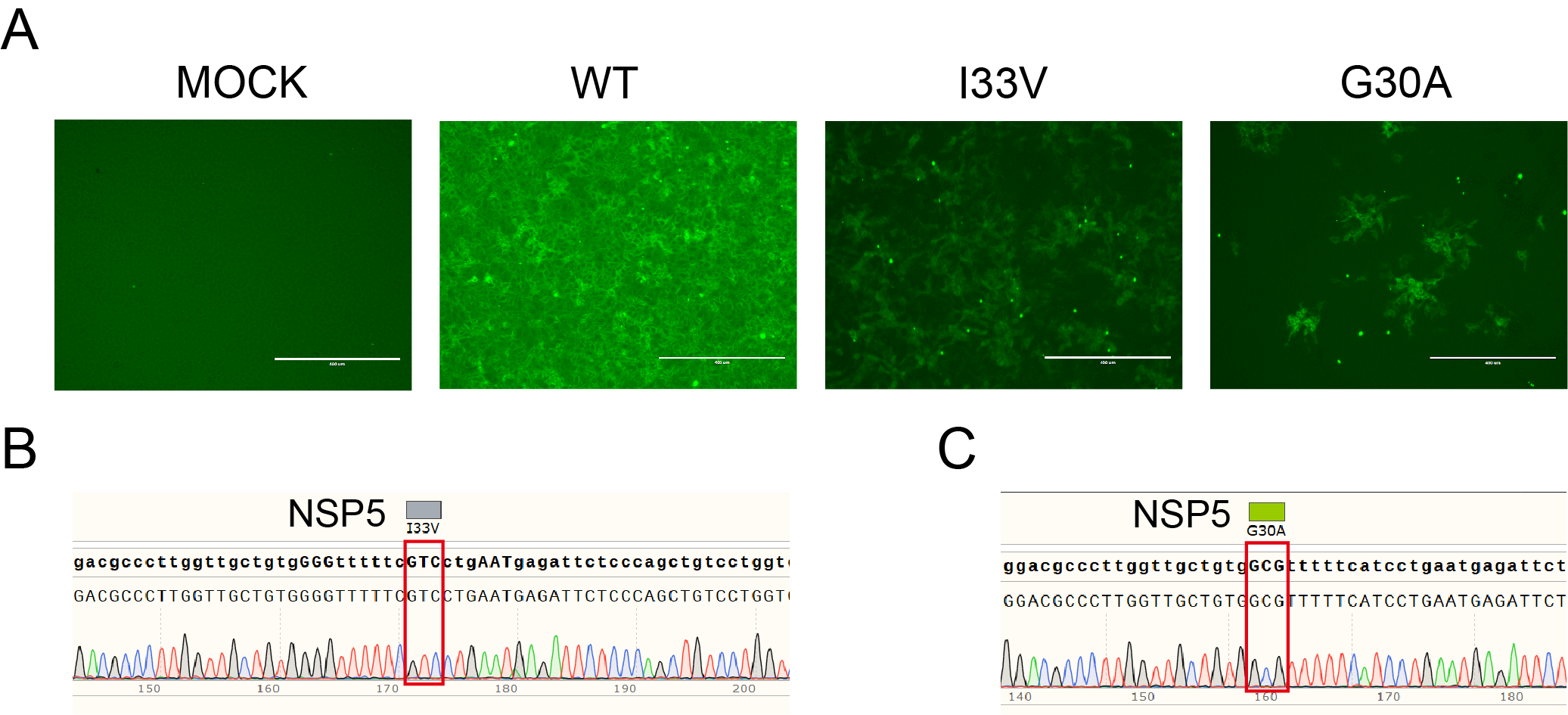

Supplement: Supplementary file 6 — Additional file 6. Rescue of PRRSV carrying the NSP5 mutations. (A) Immunofluorescence assay (IFA) of rescued PRRSV. Harvested transfected cells were infected with Marc-145 cells. Cells were stained with anti-M antibodies (Green). Scale bars = 50 μm. (B) DNA sequencing of PRRSV cDNA clones of wild-type, I33V and G30A. [file 13567_2025_1636_MOESM6_ESM.tif]

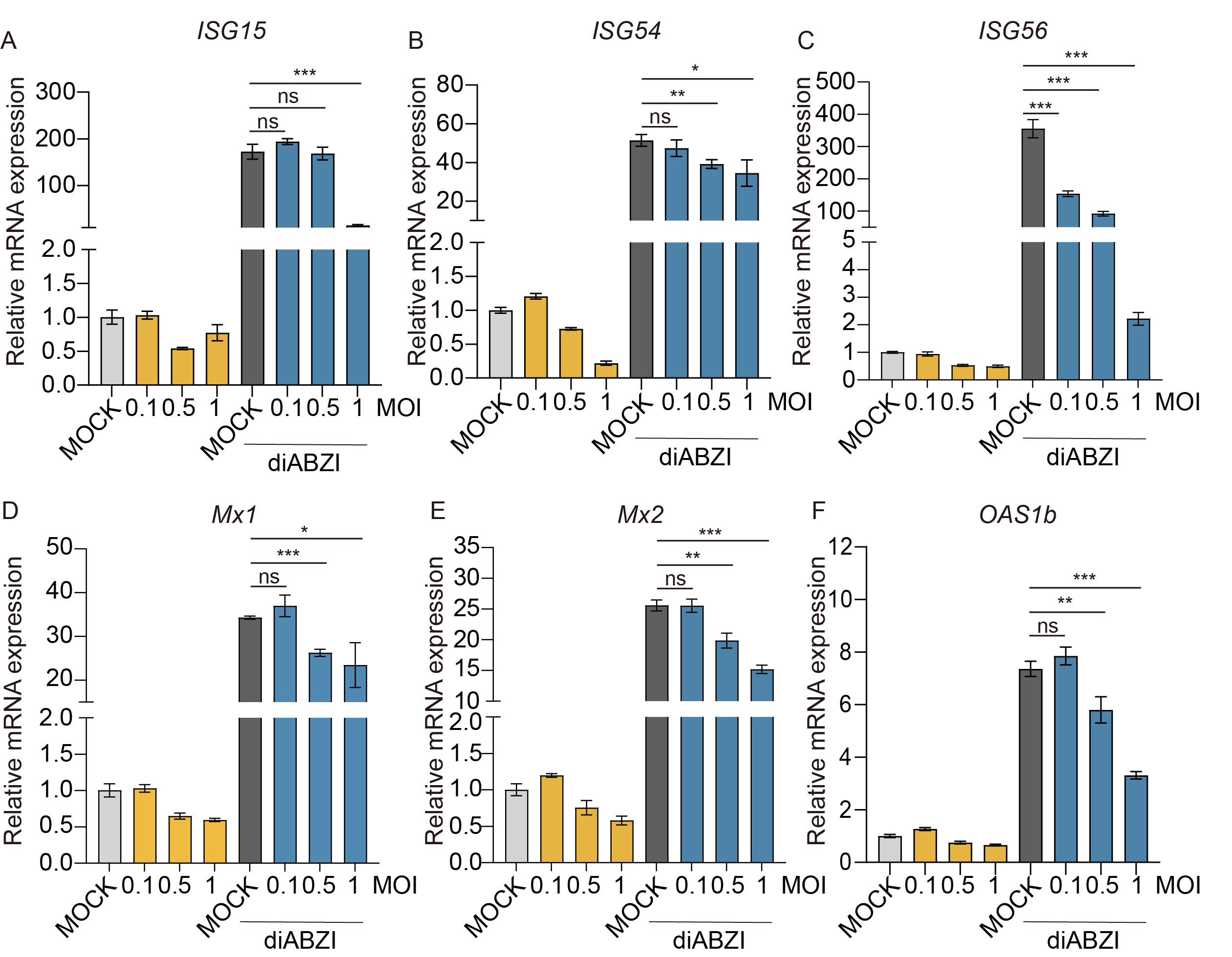

Supplement: Supplementary file 7 — Additional file 7. Quantification of ISG mRNA levels following PRRSV infection. (A to F) PAMs were infected with PRRSV at different MOIs for 24 h. The relative mRNA abundance of ISG15, ISG54, ISG56, Mx1, Mx2 and OAS1b was measured via qPCR, normalized against GAPDH, and compared to the uninfected MOCK group. The p value of less than 0.05 was considered statistically significant. * for p < 0.05, ** for p < 0.01, *** for p < 0.001, ns for not significant. [file 13567_2025_1636_MOESM7_ESM.tif]

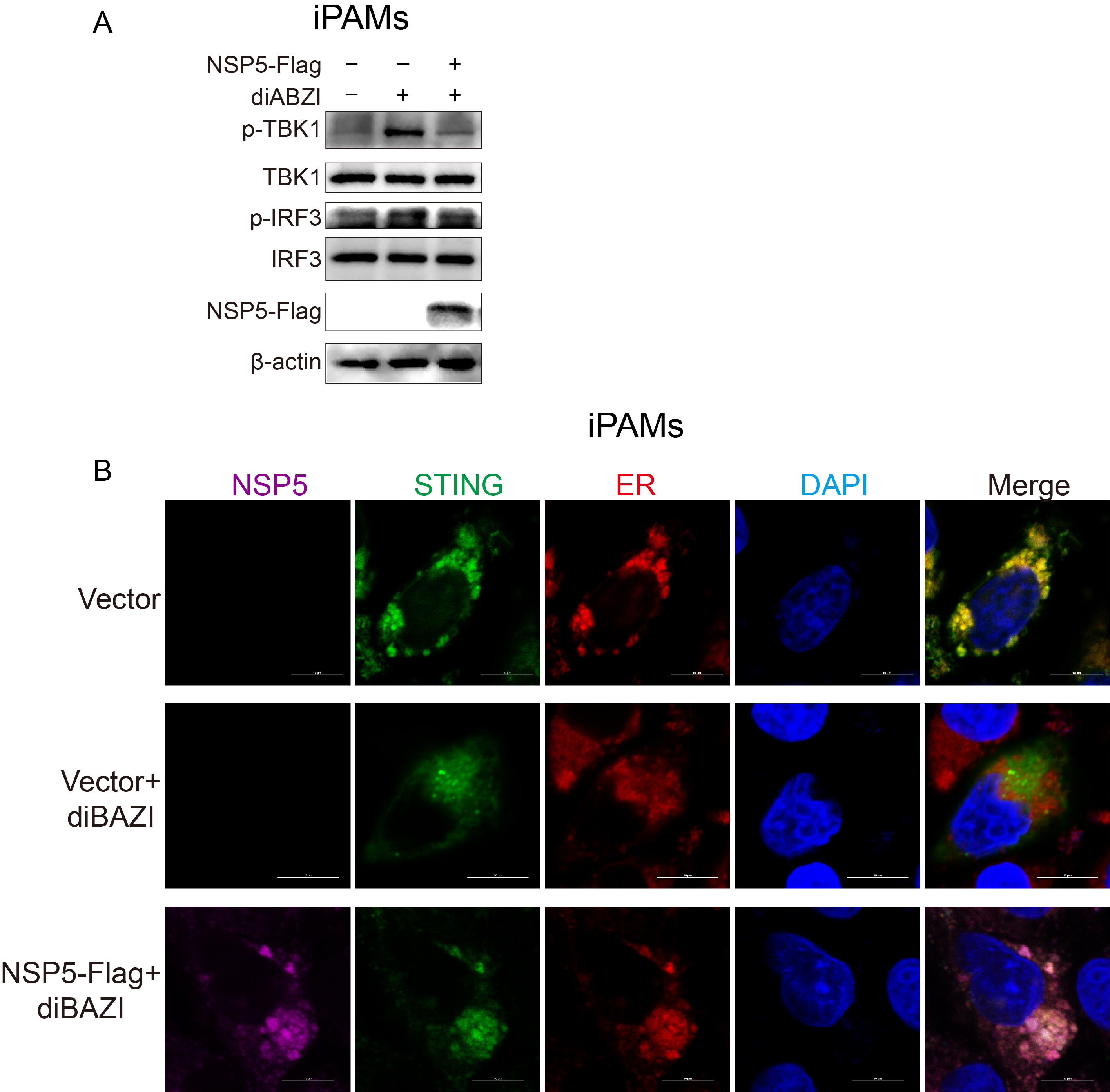

Supplement: Supplementary file 8 — Additional file 8. PRRSV NSP5 blocks STING trafficking. (A) iPAMs were transfected with a plasmid encoding NSP5-Flag for 24 h with or without diABZI. Cell lysates were analyzed by western blotting for p-TBK1, TBK1, p-IRF3, IRF3, Flag, and β-actin. (B) iPAMs were co-transfected with pcDNA3.1-NSP5-Flag or pcDNA3.1 and pcDNA3.1-STING-HA for 24 h with or without diABZI. The cells were incubated with anti-HA (green) and anti-Flag (pink) and stained with ER-specific fluorescent dye (red) and DAPI (blue) for confocal microscopy analysis. Scale bars = 10 μm. [file 13567_2025_1636_MOESM8_ESM.tif]
